# miR‐30d Attenuates Pulmonary Arterial Hypertension via Targeting MTDH and PDE5A and Modulates the Beneficial Effect of Sildenafil

**DOI:** 10.1002/advs.202407712

**Published:** 2024-08-29

**Authors:** Xuchun Liang, Jingwen Zhou, Hongyun Wang, Ziyi Zhang, Mingming Yin, Yujiao Zhu, Lin Li, Chen Chen, Meng Wei, Meiyu Hu, Cuimei Zhao, Jianhua Yao, Guoping Li, Anh‐Tuan Dinh‐Xuan, Junjie Xiao, Yihua Bei

**Affiliations:** ^1^ Institute of Geriatrics (Shanghai University), Affiliated Nantong Hospital of Shanghai University (The Sixth People's Hospital of Nantong) and School of Life Science Shanghai University Nantong 226011 China; ^2^ Joint International Research Laboratory of Biomaterials and Biotechnology in Organ Repair (Ministry of Education) Shanghai University Shanghai 200444 China; ^3^ Cardiac Regeneration and Ageing Lab Institute of Cardiovascular Sciences Shanghai Engineering Research Center of Organ Repair School of Medicine Shanghai University Shanghai 200444 China; ^4^ Department of Cardiology Shanghai Tongji Hospital Tongji University School of Medicine Shanghai 200065 China; ^5^ Department of Cardiology Tenth People's Hospital School of Medicine Tongji University Shanghai 200090 China; ^6^ Department of Cardiology Shigatse People's Hospital Tibet 857000 China; ^7^ Cardiovascular Division of the Massachusetts General Hospital and Harvard Medical School Boston MA 02114 USA; ^8^ Lung Function & Respiratory Physiology Units Department of Respiratory Physiology and Sleep Medicine Cochin & George Pompidou Hospitals Assistance Publique‐Hôpitaux de Paris (APHP) Centre University Paris Cité Paris 75014 France

**Keywords:** miR‐30d, MTDH, PDE5A, pulmonary arterial hypertension, pulmonary arterial smooth muscle cell, sildenafil

## Abstract

Pulmonary arterial hypertension (PAH) is associated with aberrant pulmonary vascular smooth muscle cell (PASMC) function and vascular remodeling. MiR‐30d plays an important role in the pathogenesis of several cardiovascular disorders. However, the function of miR‐30d in PAH progression remained unknown. Our study shows that circulating miR‐30d level is significantly reduced in the plasma from PAH patients. In miR‐30d transgenic (TG) rats, overexpressing miR‐30d attenuates monocrotaline (MCT)‐induced pulmonary hypertension (PH) and pulmonary vascular remodeling. Increasing miR‐30d also inhibits platelet‐derived growth factor‐bb (PDGF‐bb)‐induced proliferation and migration of human PASMC. Metadherin (MTDH) and phosphodiesterase 5A (PDE5A) are identified as direct target genes of miR‐30d. Meanwhile, nuclear respiratory factor 1 (NRF1) acts as a positive upstream regulator of miR‐30d. Using miR‐30d knockout (KO) rats treated with sildenafil, a PDE5A inhibitor that is used in clinical PAH therapies, it is further found that suppressing miR‐30d partially attenuates the beneficial effect of sildenafil against MCT‐induced PH and vascular remodeling. The present study shows a protective effect of miR‐30d against PAH and pulmonary vascular remodeling through targeting MTDH and PDE5A and reveals that miR‐30d modulates the beneficial effect of sildenafil in treating PAH. MiR‐30d should be a prospective target to treat PAH and pulmonary vascular remodeling.

## Graphical abstract

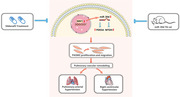

## 1 Introduction

Pulmonary arterial hypertension (PAH) is a cardiopulmonary disorder without effective treatment^[^ ^1^ ^]^ and characterized by high pulmonary artery pressure and pulmonary vascular remodeling, which ultimately causes right ventricular dysfunction.^[^ ^2^ ^]^ High mortality and poor prognosis are hallmarks of PAH,^[^ ^3^ ^]^ additional studies are needed to extend the knowledge of PAH pathologies and to develop novel therapies for PAH. At the cellular level, abnormal changes including excess pulmonary arterial smooth muscle cell (PASMC) proliferation and matrix deposition and invasion of the lumen by fibroblasts contribute to PAH. Notably, excess proliferation and migration of PASMC is a leading feature of PAH.^[^ ^4^ ^]^ Suppressing the phenotype switch or remodeling of PASMC is effective for attenuating PAH even though the underlying molecular mechanism remains unelucidated.^[^ ^5^ ^]^

PAH is a highly morbid disease with few specific therapies mainly targeting the nitric oxide, prostacyclin, and endothelin pathways.^[^ ^6^ ^]^ Most current therapies are effective to target endothelial dysfunction and improve symptoms of PAH, however, therapies that inhibit pulmonary vascular proliferation and remodeling are still lacking.^[^ ^7^ ^.^ ^8^ ^]^ Actually, the regulatory role of microRNAs (miRNA, miR) in the development of PAH attracted a growing attention owing to their regulating effect in proliferation, apoptosis, and migration of smooth muscle cells (SMC)^[^ ^9^, ^10^, ^11^ ^]^ as well as their role in the development of PAH.^[^ ^12^, ^13^ ^]^ Inhibition of miR‐17 was previously reported to improve lung and heart function and inhibit proliferation of PASMC.^[^ ^14^ ^]^ Recently, miR‐138‐5p inhibition has been shown to attenuate PH and vascular remodeling with restoration of pulmonary KCNK3 and SLC45A3 expression.^[^ ^15^ ^]^ However, more studies are needed to verify the mechanism of miRNAs in treating PAH as well as their potency for drug development.

MiR‐30d, one member of miR‐30 family plays an important role in the pathogenesis of cardiovascular disorders such as regulating myocardial infarction,^[^ ^16^ ^]^ ventricular remodeling,^[^ ^17^ ^]^ and cardiomyocyte pyroptosis.^[^ ^18^ ^]^ As a circulating miRNA enriched in blood, miR‐30d plays crucial role in regulating cell proliferation and migration such as breast cancer,^[^ ^19^ ^]^ colon cancer^[^ ^20^ ^]^ and non‐small cell lung cancer.^[^ ^21^ ^]^ We have previously shown that miR‐30d plays a cardioprotective role, inhibiting the proliferation and activation of cardiac fibroblasts and protecting mice against cardiac remodeling,^[^ ^17^ ^]^ and whose expression in human plasma is associated with beneficial response to resynchronization treatment in heart failure.^[^ ^22^ ^]^ However, the role and underlying mechanism of miR‐30d in PAH and whether miR‐30d regulates vascular proliferation and remodeling remains largely unclear.

In the present study, we examined the circulating miR‐30d level in human plasma samples from patients with PAH, and assessed the effect of miR‐30d overexpression and knockout in monocrotaline (MCT)‐induced PH rat model in vivo and in platelet‐derived growth factor (PDGF)‐induced human pulmonary artery smooth muscle cell (hPASMC) proliferation model in vitro. In addition, we identified the upstream regulator and downstream targets of miR‐30d. We show that miR‐30d is significantly reduced in plasma from PAH patients and has protective effect against PAH and pulmonary vascular remodeling through targeting metadherin (MTDH) and phosphodiesterase 5A (PDE5A), and identify nuclear respiratory factor 1 (NRF1) as an upstream regulator of miR‐30d. Moreover, we demonstrate that miR‐30d modulates the protective effect of sildenafil, a PDE5A inhibitor currently used in clinical practice, against PAH and vascular remodeling.

## 2 Results

### 2.1 MiR‐30d is Reduced in Clinical Human Plasma and Experimental Lung Tissues under Pulmonary Arterial Hypertension

**Figure 1:**
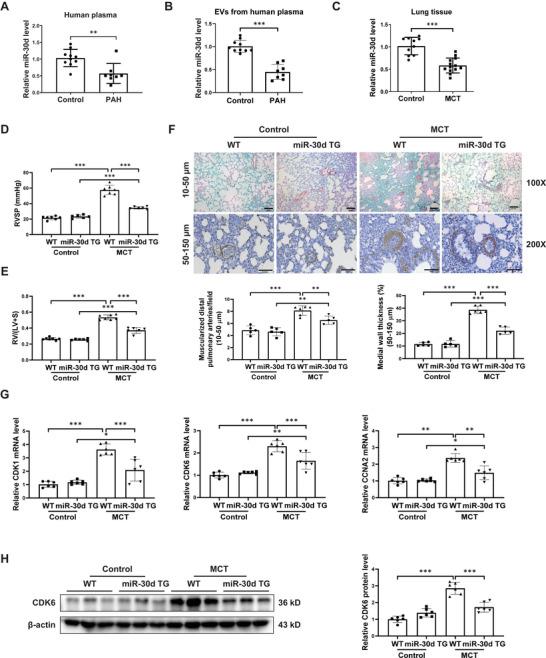
miR‐30d overexpression attenuates pulmonary hypertension and vascular remodeling. (A and B) Relative miR‐30d expression level in plasma samples (**A**) and in plasma‐derived extracellular vesicles (EVs) (**B**) from patients with idiopathic pulmonary arterial hypertension (PAH) versus healthy controls (n = 10 vs 8). C) Relative miR‐30d expression level in lung tissues of rats with monocrotaline (MCT)‐induced pulmonary hypertension (PH) (n = 10‐13). (D and E) Right ventricular systolic pressure (RVSP) (**D**) and Fulton index (RV/(LV+S) ratio) (**E**) were assessed (n = 6‐7). F) Immunohistochemical staining with α‐SMA antibody for analysis of the number of muscularized distal pulmonary arteries (10–50 µm in diameter, scale bar = 20 µm) and the medial wall thickness of pulmonary arteries (50–150 µm in diameter, scale bar = 100 µm) (n = 5‐6). G) qRT‐PCR for *CDK1*, *CDK6*, and *CCNA2* mRNA levels in lung tissues (n = 6). H) Western blot for CDK6 in lung tissues (n = 6). Data are shown as means ± SD. Data between 2 groups were compared by Mann‐Whitney U test for (**A**) and by independent‐sample two‐tailed Student's *t*‐test for (**B**) and (**C**). Data among 4 groups were compared by robust two‐way ANOVA test followed by post‐hoc pairwiseMedianTest using the rcompanion package for *CCNA2* in (**G**), and by two‐way ANOVA test followed by Tukey post hoc test for data in other figures. *P < 0.05; **P < 0.01; ***P < 0.001.

MiR‐30d, an abundant circulating miRNA, plays an important role in the pathogenesis of several cardiovascular disorders. In the present study, we collected plasma samples from patients with idiopathic PAH (n = 8) compared to healthy controls (n = 10), with their clinical characteristics shown in Table S2 (Supporting Information). All PAH patients received right heart catheterization and the mPAP was 47.88 ± 12.32 mmHg. We first observed that miR‐30d expression level was significantly reduced in the plasma as well as in the circulating EVs from patients with PAH (**Figure** 1), indicating a possible regulatory role of miR‐30d in the development of human PAH. To further investigate the alteration and function of miR‐30d in response to PAH, we established MCT‐induced PH rat model and examined the expression of miR‐30d in lung tissues. Our data showed that the expression of miR‐30d was also significantly reduced in lung tissues from PH rats (Figure 1C). Thus, miR‐30d is reduced in clinical PAH human blood samples and MCT‐induced PH model, prompting us to further investigate its function and mechanism in PAH.

### 2.2 MiR‐30d Overexpression Protects against MCT‐Induced PH and RV Hypertrophy

We first utilized miR‐30d TG rats to study the role of miR‐30d overexpression in PH. The overexpression efficiency of mature miR‐30d in lungs was shown in Figure S1A (Supporting Information), demonstrating about threefold change in miR‐30d TG rats compared to WT rats (Figure S1A, Supporting Information). As PAH can eventually lead to RV failure,^[^ ^23^ ^]^ we also examined the content of miR‐30d in RV tissues. As shown in Figure S1B (Supporting Information), miR‐30d TG rats also had marked increase of miR‐30d expression in RV tissues. Compared to WT rats, higher level of miR‐30d attenuated MCT‐induced increase of RVSP and RV/(LV+S) ratio (Figure 1D,E; Figure S1C, Supporting Information). We further examined cross‐sectional myocardial area and the hypertrophic markers and found a hypertrophy of the RV myocardium with increased expressions of *ANP* and *BNP* in MCT‐injected WT rats, whose increases were however reversed by miR‐30d overexpression (Figure S1D,E, Supporting Information). These data indicate that miR‐30d overexpression is effective to protect against MCT‐induced PH and RV hypertrophy.

### 2.3 MiR‐30d Overexpression Attenuates Pulmonary Arterial Remodeling

Though PH has different subcategories, abnormal vascular proliferation and remodeling in the small distal pulmonary arteries is a common characterization.^[^ ^4^ ^]^ To assess the pulmonary arterial remodeling in lung tissues, small distal pulmonary arteries (10–50 µm) and larger pulmonary arteries (50–150 µm) were profiled using immunohistochemistry (IHC) staining by anti‐α‐SMA antibody. Our data showed that MCT‐induced PH model had a marked increase of muscularized distal pulmonary arteries as well as medial wall thickness (Figure 1F). Overexpression of miR‐30d reduced the number of muscularized distal pulmonary arteries compared to WT rats. In addition, the medial wall thickness of miR‐30d TG rats is less than that of WT rats in the MCT model (Figure 1F). Cyclin‐dependent kinases (CDKs), such as CDK1 and CDK6, as well as cyclin A2 (CCNA2) play a critical role in SMC proliferation.^[^ ^24^, ^25^, ^26^, ^27^ ^]^ Compared to WT rats, miR‐30d TG rats had a lower expression of *CDK1*, *CDK6* and *CCNA2* in the MCT model (Figure 1G). Western blot showed a consistent increase of CDK6 protein level induced by MCT, which was significantly reversed by miR‐30d overexpression (Figure 1H). Collectively, these data demonstrate that miR‐30d overexpression attenuates pulmonary arterial remodeling via inhibiting vascular proliferation.

### 2.4 Overexpressing miR‐30d inhibits hPASMC Proliferation and Migration

**Figure 2:**
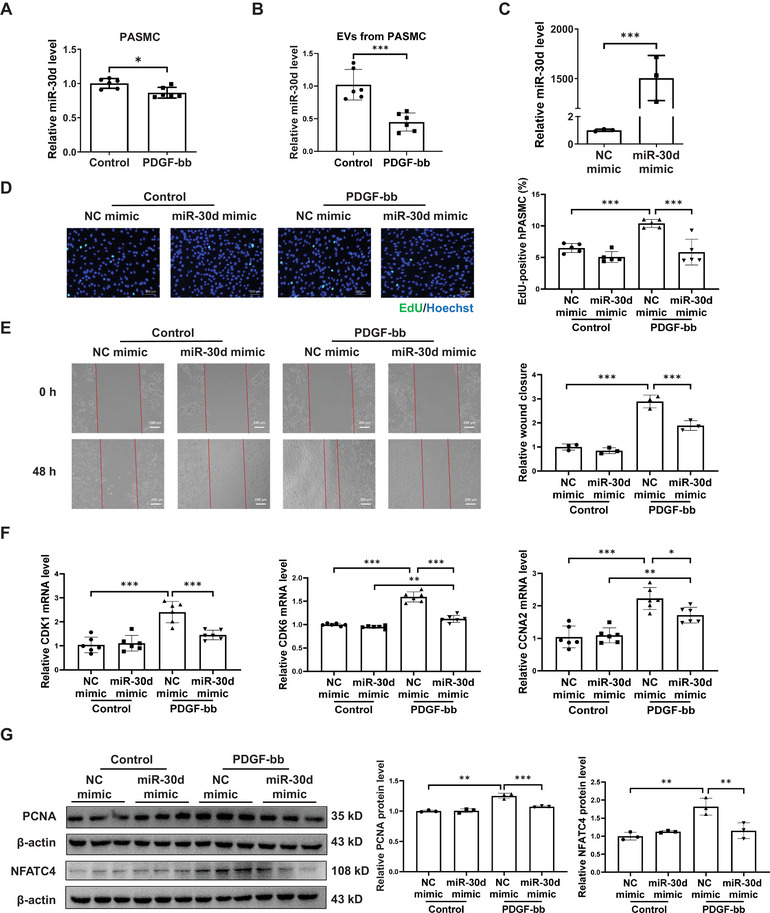
Overexpressing miR‐30d inhibits hPASMC proliferation and migration. A) Relative miR‐30d expression level in human pulmonary arterial smooth muscle cells (hPASMC) stressed with platelet‐derived growth factor‐bb (PDGF‐bb) for 48h (n = 6). B) Relative miR‐30d expression level in extracellular vesicles (EVs) isolated from the culture medium of rat PASMC after treatment with PDGF‐bb or control vehicles for 48h (n = 6). C) The overexpression efficiency of miR‐30d mimic in hPASMC (n = 3). D) Representative images and quantification of EdU/Hoechst staining of miR‐30d mimic or negative control (NC) transfected hPASMC under PDGF‐bb stress (n = 5). Scale bar = 200 µm. E) Scratch wound healing assay was performed to investigate the effect of miR‐30d mimic on hPASMC migration. Representative images and relative wound closure of miR‐30d mimic or NC transfected hPASMC under PDGF‐bb stress (n = 3). Scale bar = 200 µm. F) qRT‐PCR for *CDK1*, *CDK6*, and *CCNA2* mRNA levels in hPASMC (n = 6). G) Western blot for PCNA and NFATC4 in hPASMC (n = 3). Data are shown as means ± SD. Data between 2 groups were compared by independent‐sample two‐tailed Student's *t*‐test for (A) to (C). Data among 4 groups were compared by two‐way ANOVA test followed by Tukey post hoc test for (D) to (G). *P < 0.05; **P < 0.01; ***P < 0.001.

To investigate the mechanism of miR‐30d resistance to PAH, an in vitro model was used with hPASMC based on the previous study.^[^ ^28^ ^]^ The hPASMC were treated with platelet‐derived growth factor‐bb (PDGF‐bb), a primary mediator in PAH‐associated pulmonary vascular remodeling. As shown in **Figure** **2**A, 30 ng ml^−1^ of PDGF‐bb treatment reduced miR‐30d expression in hPASMC. Meanwhile, EVs derived from rat PASMC also had lower miR‐30d expressions in the PDGF‐bb group (Figure 2B). To clarify the function of miR‐30d in PDGF‐induced proliferation, miR‐30d mimic was used to efficiently transfect hPASMC (Figure 2C). Overexpressing miR‐30d significantly reduced PDGF‐induced hPASMC proliferation (Figure 2D). We also performed wound healing experiment to evaluate the function of miR‐30d in hPASMC migration. At basal condition, overexpressing miR‐30d did not alter wound closure, however, it significantly attenuated PDGF‐induced hPASMC migration (Figure 2E). We then evaluated the effect of miR‐30d on cell proliferation and migration markers, showing that overexpressing miR‐30d downregulated *CDK1*, *CDK6*, and *CCNA2* (Figure 2F) and reduced the protein levels of PCNA and NFATC4 in PDGF‐stressed hPASMC (Figure 2G). These data strengthen the hypothesis that overexpressing miR‐30d inhibits hPASMC proliferation and migration.

In addition to PDGF‐bb treatment, the function of miR‐30d was also determined in hPASMC under hypoxia condition; although miR‐30d was found to be increased in the hypoxic PASMC (Figure S2A, Supporting Information), overexpressing miR‐30d was still protective to inhibit hPASMC proliferation and migration (Figure S2B–D, Supporting Information). Meanwhile, increasing miR‐30d also reduced the proliferation of human pulmonary arterial endothelial cells (hPAEC) and inhibited their resistance to apoptosis under hypoxic stress (Figure S3, Supporting Information). Collectively, these data support that miR‐30d functions to inhibit pulmonary vascular remodeling upon the pathological stress of PAH.

### 2.5 Ablation of miR‐30d does not Aggravates PH

To explore the effect of miR‐30d deficiency on PAH, we utilized whole‐body miR‐30d KO rats for MCT‐induced PH model in vivo. MiR‐30d KO rats had obvious ablation of miR‐30d expression in lung tissues (Figure S4A, Supporting Information). We observed that miR‐30d deficiency altered RVSP and RV/(LV+S) ratio neither in control nor in MCT group (Figure S4B–D, Supporting Information), indicating that suppressing miR‐30d did not further aggravate PH in vivo. Then, we transfected miR‐30d inhibitor in hPASMC for loss‐of‐function experiment (Figure S4E, Supporting Information). In PDGF‐stressed hPASMC, there was no difference of EdU‐positive hPASMC between NC inhibitor and miR‐30d inhibitor group (Figure S4F, Supporting Information). MiR‐30d inhibitor did not alter PDGF‐induced wound closure (Figure S4G, Supporting Information) or the expressions of *CDK1*, *CDK6* and *CCNA2* in hPASMC (Figure S4H, Supporting Information), either. It can be hypothesized that the pathological changes of PH experimental model were evident that cannot be further aggravated by miR‐30d suppression.

### 2.6 MTDH and PDE5A are Target Genes of miR‐30d

**Figure 3:**
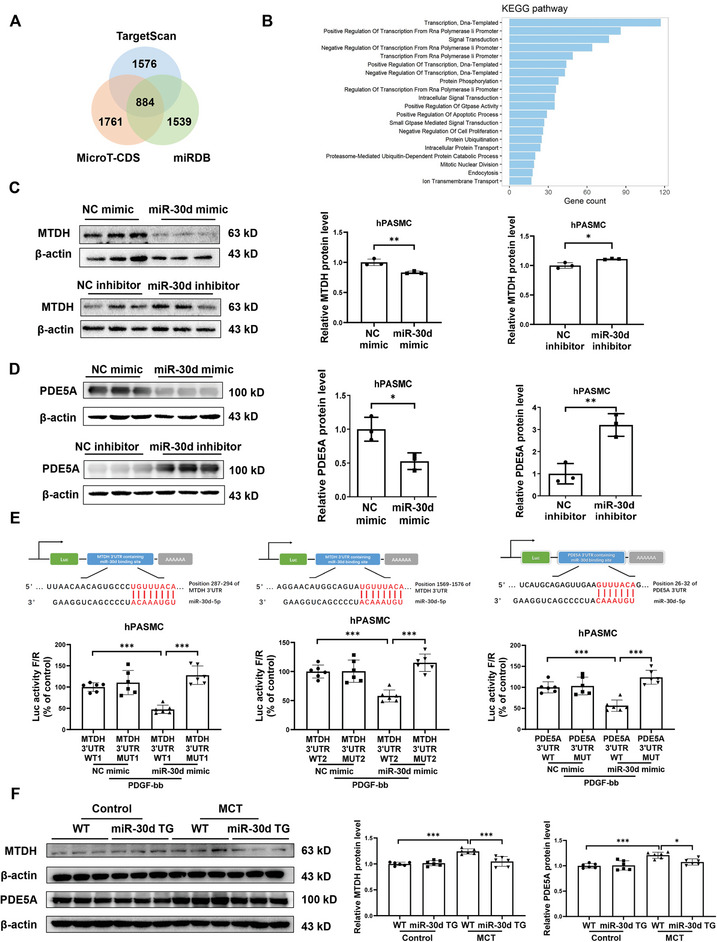
MTDH and PDE5A are downstream targets of miR‐30d. A) The intersection of TargetScan, MicroT‐CDS, and miRDB for predicting target genes of miR‐30d. B) KEGG pathway of the enriched potential target genes of miR‐30d. (C and D) Western blot for MTDH (C) and PDE5A (D) in human pulmonary arterial smooth muscle cells (hPASMC) transfected with miR‐30d mimic or inhibitor and negative control (NC) (n = 3). E) Luciferase reporter assay was performed in hPASMC treated with platelet‐derived growth factor‐bb (PDGF‐bb), simultaneously transfected with the recombinant plasmids containing wild type 3’‐UTR region of MTDH or PDE5A or relevant mutant sequence along with miR‐30d mimic or NC mimic (n = 6). F) Western blot for MTDH and PDE5A in lung tissues from wild type (WT) or miR‐30d transgenic (TG) rats injected with monocrotaline (MCT) or control (n = 6). Data are shown as means ± SD. Data between 2 groups were compared by independent‐sample two‐tailed Student's *t*‐test for (C) and (D). Data among 4 groups were compared by two‐way ANOVA test followed by Tukey post hoc test for (E) and (F). *P < 0.05; **P < 0.01; ***P < 0.001.

To identify the downstream targets of miR‐30d mediating its protective role in PAH, we collected the predicted targets of miR‐30d through bioinformatic websites, including TargetScan, MicroT‐CDS, and miRDB. The intersection of these three web sources showed that 884 genes were potential targets of miR‐30d (**Figure** **3**A). The enriched KEGG pathway of these genes included ‘positive regulation of apoptotic process’ and ‘negative regulation of cell proliferation’ (Figure 3B). We then examined the genes involved in these two pathways and assessed their mRNA levels in response to miR‐30d regulation. Two genes in response to both miR‐30d overexpression and knockdown were identified, namely metadherin (MTDH) and phosphodiesterase 5A (PDE5A). The mRNA levels of *MTDH* and *PDE5A* were downregulated by miR‐30d mimic, while upregulated by miR‐30d inhibitor in hPASMC (Figure S5A,B, Supporting Information). To further verify the relationship, we evaluated the protein content of MTDH and PDE5A and their binding sites with miR‐30d. Overexpressing miR‐30d significantly downregulated MTDH and PDE5A at protein level, whose expressions were upregulated when inhibiting miR‐30d in hPASMC (Figure 3C,D). Based on bioinformatic analysis, miR‐30d predicted binding sites in the 3’‐UTR regions of MTDH and PDE5A were found (Figure 3E). According to the predicted locations, we constructed luciferase recombinant plasmids containing MTDH‐3’UTR (position: 287–294 and 1569–1576) or mutant control, as well as PDE5A‐3’UTR (position: 26–32) or mutant control, and then co‐transfected them with miR‐30d mimic in 293T cells. Luciferase reporter assays indicated that miR‐30d mimic significantly reduced the luciferase activity of 3’‐UTR in MTDH and PDE5A but no change was seen in mutant control (Figure S5C, Supporting Information). Furthermore, luciferase reporter assays were conducted in PDGF‐stressed hPASMC which consistently demonstrated the direct binding of miR‐30d to the 3’‐UTR regions of MTDH and PDE5A (Figure 3E).

We then determined the regulation of miR‐30d on MTDH and PDE5A expressions in the in vivo PH experimental model. Interestingly, we observed that miR‐30d knockout was not able to further increase MTDH and PDE5A expression levels in lung tissues (Figure S5D, Supporting Information), which might at least in part explain the reason why suppressing miR‐30d could not further aggravate PH in vivo. However, overexpression of miR‐30d significantly reduced the expression of MTDH and PDE5A in MCT‐induced PH rat lung tissues (Figure 3F; Figure S5E, Supporting Information), indicating the inhibitory effect of miR‐30d on MTDH and PDE5A expressions in vivo.

### 2.7 MiR‐30d Inhibits hPASMC Proliferation and Migration through Targeting MTDH and PDE5A

**Figure 4:**
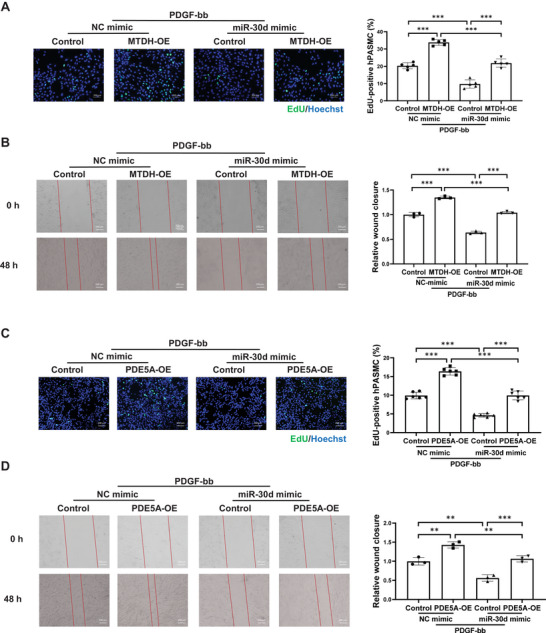
miR‐30d inhibits hPASMC proliferation and migration through targeting MTDH and PDE5A. A and C) EdU‐positive cells in MTDH (A) or PDE5A (C) overexpressing and/or miR‐30d overexpressing human pulmonary arterial smooth muscle cells (hPASMC) under PDGF‐bb stress (n = 5‐6). Scale bar = 200 µm. B and D) Wound closure of scratch experiment in MTDH (B) or PDE5A (D) overexpressing and/or miR‐30d overexpressing hPASMC under PDGF‐bb stress (n = 3). Scale bar = 200 µm. Data are shown as means ± SD. Data among 4 groups were compared by two‐way ANOVA test followed by Tukey post hoc test for (A) to (D). **P < 0.01; ***P < 0.001.

To further elucidate the downstream targets of miR‐30d in regulating hPASMC functions, we evaluated MTDH and PDE5A expressions in PDGF‐stressed hPASMC and performed function‐rescue experiments. Our results showed that both MTDH and PDE5A were upregulated in PDGF‐treated hPASMC, while miR‐30d mimic significantly downregulated MTDH and PDE5A mRNA levels in both PDGF‐treated hPASMC or control hPASMC (Figure S6A,B, Supporting Information). To further study whether MTDH or PDE5A inhibition mediates the protective role of miR‐30d, hPASMC were cultured and efficiently transfected with MTDH‐overexpressing (OE) or PDE5A‐OE plasmids (Figure S6C,D, Supporting Information), complemented with miR‐30d mimic transfection in PDGF‐induced model, respectively. Function‐rescue experiments showed that overexpression of miR‐30d‐induced protective effects against hPASMC proliferation and migration were attenuated by MTDH‐OE or PDE5A‐OE plasmids (**Figure** **4**A–D). Moreover, simultaneous overexpression of MTDH and PDE5A could not have additive effect when compared to their individual effect in attenuating the function of miR‐30d mimic (Figure S6E,F, Supporting Information), suggesting that MTDH and PDE5A did not totally act independently from each other in regulating hPASMC functions. Taken together, these results indicate that miR‐30d inhibits hPASMC proliferation and migration through targeting MTDH and PDE5A.

### 2.8 NRF1 Acts as an Upstream of miR‐30d in Regulating hPASMC Functions

**Figure 5:**
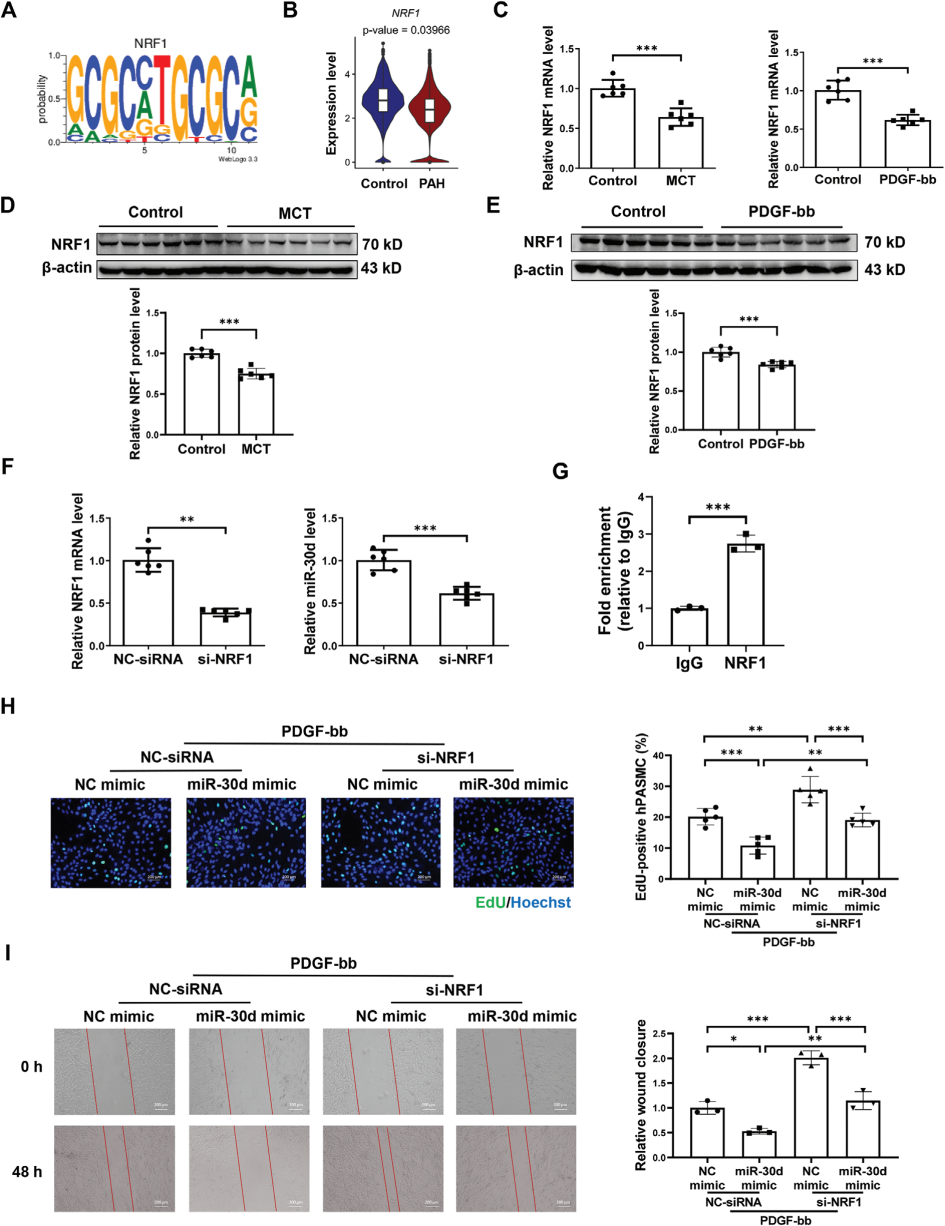
NRF‐1 is upstream regulator of miR‐30d. A) Predicting NRF1 as potential upstream regulators of miR‐30d through DIANA TOOLS (miRGen v.3). B) According to the online single‐cell RNA sequencing data (GEO: GSE210248) of the isolated pulmonary arteries from idiopathic pulmonary arterial hypertension (PAH) patient lungs versus donor lungs, comparison of NRF1 expressions in the cluster of pulmonary arterial smooth cells (PASMC) was demonstrated. C) qRT‐PCR analysis for *NRF1* in lung tissues from monocrotaline (MCT)‐induced pulmonary hypertension (PH) model and in PDGF‐treated human PASMC (n = 6). D and E) Western blot for NRF1 in lung tissues from MCT‐induced PH model (D) and in PDGF‐treated human PASMC (E) (n = 6). F) qRT‐PCR for *NRF1* and miR‐30d in human PASMC transfected with NRF1 siRNA (si‐NRF1) or negative control (NC‐siRNA) (n = 6). G) ChIP assay to determine the enrichment of NRF1 in the promoter region of miR‐30d using rat PASMC (n = 3). H) EdU‐positive cells in human PASMC co‐transfected with miR‐30d mimic and/or si‐NRF1 under PDGF‐bb stress (n = 5). Scale bar = 200 µm. I) Wound closure of scratch experiment in human PASMC co‐transfected with miR‐30d mimic and/or si‐NRF1 under PDGF‐bb stress (n = 3). Scale bar = 200 µm. Data are shown as means ± SD. Data between 2 groups were compared by independent‐sample two‐tailed Student's *t*‐test for (C) to (E) and (G), and for miR‐30d in (F), and by Mann‐Whitney U test for *NRF‐1* in (F). Data among 4 groups were compared by two‐way ANOVA test followed by Tukey post hoc test for (H) and (I). *P < 0.05; **P < 0.01; ***P < 0.001.

As miR‐30d is reduced in PAH model, the upstream regulation of miR‐30d is important to sort out. Therefore, the potential upstream of miR‐30d was predicted through DIANA TOOLS (miRGen v.3). As shown in **Figures** **5**A and S7A (Supporting Information), NRF1 and CREB1 were identified to be possibly involved in interacting with the promoter region of miR‐30d. We then tried to analyze the online single cell RNA sequencing data (accession number GSE210248) of the isolated pulmonary arteries from idiopathic PAH patient lungs versus donor lungs that have been previously published and uploaded to the National Center of Biotechnology Information Gene Expression Omnibus (GEO) database.^[^ ^29^ ^]^ Using the uniform manifold approximation and projection (UMAP) cluster analysis, we totally identified 27 cell clusters in both control group and PAH group. Furthermore, a cell cluster with obvious expressions of *MYH11*, *TAGLN*, and *CNN1* was identified for PASMC. We found that *NRF1* was significantly downregulated in the PASMC of PAH group compared to control group (*P* = 0.03966) (Figure 5B); while *CREB1* was not significantly regulated in the PAH group (*P* = 0.14909) (Figure S7B, Supporting Information).

To further verify the actual response of NRF1 and CREB1 to PAH, their mRNA expressions were assessed in the present study. Both NRF1 and CREB1 were decreased in MCT‐induced PH model or PDGF‐stressed hPASMC (Figure 5C–E; Figure S7C, Supporting Information), which was consistent to the expression change of miR‐30d in these models. Suppressing NRF‐1 but not CREB1 induced a reduction of miR‐30d level in hPASMC (Figure 5F; Figure S7D, Supporting Information). ChIP assay confirmed a direct binding of NRF1 to the promoter region of miR‐30d (Figure 5G). Interestingly, in consistent with the upregulation of miR‐30d in hypoxic hPASMC, *NRF‐1* was also upregulated upon hypoxic stress in hPASMC while *CREB1* expression was not altered (Figure S8, Supporting Information). Collectively, these data indicate that NRF1 acts as a positive transcriptional regulator of miR‐30d in PAH. Thereby, we further performed a co‐transfection experiment with NRF1‐siRNA/miR‐30d mimic or their relative NC under PDGF‐bb stress. Knockdown of NRF‐1 exacerbated PDGF‐induced hPASMC proliferation and migration, while these phonotypes were rescued by miR‐30d mimic (Figure 5H,I). These data suggest that NRF‐1 acts as an upstream of miR‐30d in PAH associated with hPASMC proliferation and migration.

### 2.9 MiR‐30d Modulates the Beneficial Effect of Sildenafil against PH

Sildenafil has been used as an effective agent in treating PAH.^[^ ^30^, ^31^ ^]^ Interestingly, we noticed that miR‐30d can directly target PDE5A, also a well‐known target of sildenafil. Thus, we were attracted by the underlying relationship between sildenafil and miR‐30d in the context of PAH. First, we observed that in PH rat model treated with or without sildenafil, both *NRF1* and miR‐30d were significantly downregulated in the lung tissues from MCT group, while reversed by sildenafil treatment (Figure S9A,B, Supporting Information). We further examined the expression of NRF1 and miR‐30d in PDGF‐stressed hPASMC treated with sildenafil, and demonstrated that PDGF‐induced downregulations of *NRF1* and miR‐30d were also significantly reversed by sildenafil treatment (Figure S9C, Supporting Information). While the downstream target genes of miR‐30d including *MTDH* and *PDE5A* were both downregulated by sildenafil treatment in PDGF‐induced hPASMC (Figure S9D, Supporting Information). These data suggest that sildenafil treatment can partly rescue miR‐30d expression in PAH probably via NRF1 activation.

**Figure 6:**
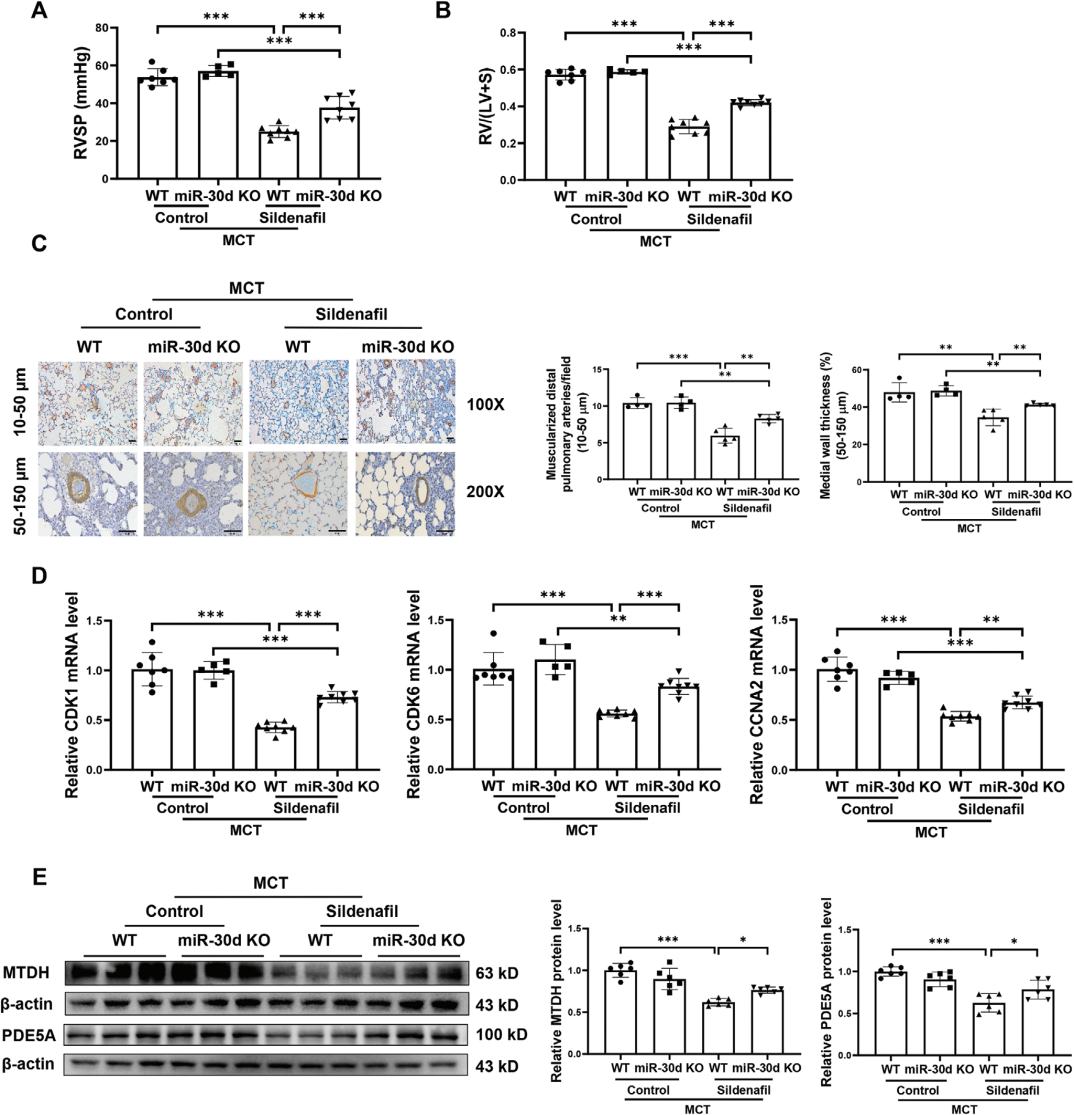
MiR‐30d modulates the beneficial effect of sildenafil against pulmonary hypertension. We utilized miR‐30d knockout (KO) rats to receive sildenafil treatment in a monocrotaline (MCT)‐induced pulmonary hypertension (PH) model. A and B) Right ventricular systolic pressure (RVSP) (A) and Fulton index (RV/(LV+S) ratio) (B) were assessed (n = 5‐7 for control‐treated MCT rats, n = 8 for sildenafil‐treated MCT rats). C) Immunohistochemical staining with α‐SMA antibody for analysis of the number of muscularized distal pulmonary arteries (10–50 µm in diameter, scale bar = 20 µm) and the medial wall thickness of pulmonary arteries (50–150 µm in diameter, scale bar = 100 µm) (n = 4‐5). D) qRT‐PCR for *CDK1*, *CDK6*, and *CCNA2* mRNA levels in lung tissues (n = 5‐7 for control‐treated MCT rats, n = 8 for sildenafil‐treated MCT rats). E) Western blot for MTDH and PDE5A protein levels in lung tissues of WT or miR‐30d KO rats in PH model treated with sildenafil or not (n = 6). Data are shown as means ± SD. Data among 4 groups were compared by robust two‐way ANOVA test followed by post‐hoc pairwiseMedianTest using the rcompanion package for medial wall thickness in (C) and for *CDK6* in (D), and by two‐way ANOVA test followed by Tukey post hoc test for data in other figures. *P < 0.05; **P < 0.01; ***P < 0.001.

Next, we used miR‐30d KO rats to perform MCT‐induced PH model with sildenafil treatment to further elucidate the regulatory relationship between sildenafil and miR‐30d in vivo. Our data showed that miR‐30d deficiency partially attenuated the protective role of sildenafil in reducing RVSP and RV/(LV+S) ratio (**Figure** **6A,B**; Figure S10A, Supporting Information). Sildenafil induced the expression of miR‐30d in both lung and RV tissues of MCT rats (Figure S10B,C, Supporting Information). Meanwhile, sildenafil treatment reduced hypertrophic marker *ANP* and *BNP* expressions in RV tissues of MCT rats, which was however partially attenuated by miR‐30d deficiency (Figure S10D, Supporting Information). To further explore whether miR‐30d is involved in the protective effect of sildenafil against vascular remodeling, we performed a series of analyses for pulmonary arterial remodeling. Sildenafil significantly reduced muscularized distal pulmonary arteries (10–50 µm) numbers and medial wall thickness of larger arteries (50–150 µm), while miR‐30d deficiency reversed these changes (Figure 6C). The reduced expression levels of *CDK1*, *CDK6*, and *CCNA2* after sildenafil treatment were also reversed by miR‐30d deficiency (Figure 6D; Figure S10E, Supporting Information). In consistent to an increased expression of miR‐30d, we also observed that sildenafil‐treated PH rats had reduced MTDH and PDE5A expressions, however these changes were attenuated in miR‐30d deficiency rats (Figure 6E). Meanwhile, function‐rescue experiments in hPASMC with sildenafil treatment and miR‐30d inhibitor transfection showed that inhibiting miR‐30d significantly attenuated sildenafil‐induced protective effects against hPASMC proliferation and migration (Figure S9E,F, Supporting Information). All these data suggest that miR‐30d modulates the protective effect of sildenafil in treating PH and vascular remodeling.

## 3 Discussion

**Figure 7:**
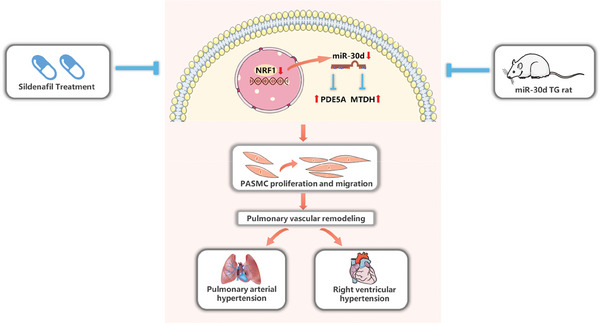
Schematic diagram illustrating the role of miR‐30d in pulmonary arterial hypertension. MiR‐30d is downregulated in pulmonary arterial hypertension (PAH) which is mainly induced by a reduced expression of NRF1. Overexpressing miR‐30d prevents PAH and pulmonary vascular remodeling and inhibits pulmonary arterial smooth muscle cell (PASMC) proliferation and migration through directly targeting metadherin (MTDH) and phosphodiesterase 5A (PDE5A). MiR‐30d, at least in part, contributes to the effect of sildenafil in treating PAH.

The present study elucidated a protective effect of miR‐30d against the development of PAH and pulmonary vascular remodeling. Circulating miR‐30d level was significantly reduced in patients with PAH. Consistently, miR‐30d expression was downregulated in lung tissues from MCT‐induced PH rat model, while overexpressing miR‐30d prevented PH and pulmonary vascular remodeling. Mechanistically, miR‐30d was effective to inhibit PASMC proliferation and migration through targeting MTDH and PDE5A. We further revealed that reduction of miR‐30d in PAH was mainly induced by a reduced expression of NRF1. Moreover, using miR‐30d KO rats treated with sildenafil, we provided compelling evidence that miR‐30d, at least in part, contributed to the effect of sildenafil in treating PAH (**Figure** **7**).

We have previously reported that miR‐30d regulates cardiac remodeling through promoting cardiomyocyte survival while inhibiting cardiac fibroblast proliferation and activation.^[^ ^17^ ^]^ Others and our previous study have also shown that miR‐30d could prevent pathological cardiac hypertrophy,^[^ ^32^ ^]^ and regulate other functions of cardiomyocytes such as apoptosis, pyroptosis, and autophagy.^[^ ^16^, ^17^, ^18^ ^]^ Interestingly, circulating miR‐30d is abundant in human blood and is associated with heart failure in patients and their response to cardiac resynchronization therapy.^[^ ^22^, ^32^ ^]^ However, the potential role of miR‐30d in PAH remained largely unclear. In the present study, we first observed that circulating miR‐30d level was markedly reduced in both human plasma samples and plasma‐derived EVs from PAH patients compared to healthy controls. Consistently, we identified significant downregulation of miR‐30d in lung tissues from MCT‐induced PH rat model and in PDGF‐stimulated hPASMC in vitro model. MiR‐30d was also found to be reduced in PASMC‐derived EVs after PDGF‐bb treatment. Functionally, overexpressing miR‐30d had significant protective effect against PH and RV hypertrophy in MCT‐induced PH model, and markedly attenuated pulmonary vascular remodeling as evidenced by reduced number of muscularized distal pulmonary arteries and reduced medial wall thickness of pulmonary arteries. Excess proliferation of PASMC, one of the primary characteristics of pulmonary vascular remodeling, is associated with active cell cycle. The CDK family members, such as CDK1 and CDK6, are associated with active cell cycle and play crucial roles in promoting PASMC proliferation.^[^ ^33^ ^]^ Therefore, inhibition of CDKs may be beneficial to attenuate PAH.^[^ ^33^ ^]^ In the present study, we further found that increasing miR‐30d significantly inhibited PDGF‐induced proliferation and migration of hPASMC in vitro. Moreover, miR‐30d could inhibit the expression of CDK1, CDK6 and CCNA2 in the lung tissues from MCT‐induced PH rat model and in PDGF‐bb stimulated hPASMC in vitro as well. In fact, miR‐30d was previously identified to regulate the proliferation and migration of several cancer cells such as nephroblastoma,^[^ ^34^ ^]^ esophageal squamous cell carcinoma^[^ ^35^ ^]^ and pituitary adenoma cell.^[^ ^36^ ^]^ Therefore, miR‐30d could be an important target in regulating proliferation‐associated diseases. Our findings of the functional role of miR‐30d in reducing PASMC proliferation and migration may potentially contribute to the protective effect of miR‐30d against PAH and pulmonary vascular remodeling. Meanwhile, we demonstrated that overexpressing miR‐30d also inhibited the proliferation of hPAEC and reduced the apoptosis‐resistance of hPAEC. According to our results, PASMC after PDGF‐bb treatment showed reduced expression of miR‐30d in PASMC and in their exosomes as well. Increasing evidence has shown the critical involvements of EVs in vascular diseases.^[^ ^37^ ^]^ Endothelial cells (EC) can interact with SMC through EVs which contribute to PH vascular remodeling.^[^ ^38^ ^]^ Indeed, it deserves further investigating the role of miR‐30d in EC in conjunction with cell‐cell interactions with PASMC.

Bioinformatic prediction and experimental verification further identified that both MTDH and PDE5A were regulated by miR‐30d in the development of PAH. We showed that miR‐30d negatively regulated MTDH and PDE5A in hPASMC and directly targeted 3’UTR of MTDH and PDE5A either with PDGF‐bb stress or not, indicating MTDH and PDE5A as direct downstream targets of miR‐30d. It is noteworthy that miR‐30d KO was not able to upregulate MTDH or PDE5A in lung tissues in vivo, which might at least in part explain the phenomenon that miR‐30d ablation did not worsen PH any further in MCT‐injected rats. However, overexpressing miR‐30d was effective to downregulate MTDH and PDE5A expressions in lung tissues from MCT‐induced PH rat model and in PDGF‐bb stimulated hPASMC in vitro.

MTDH, an oncogene associated with active cell proliferation, has been known to be involved in regulating cancer development.^[^ ^39^, ^40^ ^]^ However, the potential role of MTDH in regulating PASMC had been largely unknown. On the other hand, PDE5A has been reported to be critically involved in cardiovascular diseases and cardiopulmonary disorders,^[^ ^41^, ^42^ ^]^ including pathological cardiac hypertrophy,^[^ ^43^ ^]^ pulmonary hypertension,^[^ ^44^ ^]^ and abdominal aortic aneurysm.^[^ ^45^ ^]^ Here in our study, we further demonstrated that overexpression of MTDH or PDE5A could block the protective effect of miR‐30d against hPASMC proliferation and migration, providing direct evidence that MTDH and PDE5A act as downstream targets mediating the functional role of miR‐30d in hPASMC. Thus, we identified two target genes of miR‐30d, including MTDH, a totally novel identified molecule involved in PASMC function, and PDE5A, a well‐known molecule that can be targeted in PAH treatment. Notably, MTDH was found to regulate inflammation,^[^ ^46^ ^]^ while alteration of inflammatory factors was not evaluated in our model, which deserves further exploration.

To further investigate the upstream regulator of miR‐30d in the development of PAH, we preliminarily identified NRF1 as a potential upstream regulator of miR‐30d via bioinformatic prediction and then performed function‐rescue experiments to verify the regulatory relationship between NRF1 and miR‐30d. NRF1 (Nuclear respiratory factor 1) is a transcription factor closely related to modulation of key metabolic genes for cell growth and development.^[^ ^47^ ^]^ One recent study showed that NRF1 attenuated the development of atherosclerosis via suppressing the proliferation and migration of vascular SMC,^[^ ^48^ ^]^ indicating a protective role of NRF1 in regulating vascular remodeling. The online single cell RNA sequencing data (GSE210248) revealed a downregulation of NRF1 in PASMC of IPAH patients. However, the role of NRF1 has never been reported in PAH. In the present study, we observed a downregulation of NRF1 in both MCT‐induced PH rat model and PDGF‐bb stimulated hPASMC and demonstrated that knockdown of NRF1 caused a downregulation of miR‐30d in hPASMC. Function‐rescue experiments further demonstrated that knockdown of NRF1 enhanced the proliferation and migration of hPASMC, which was however reversed by miR‐30d overexpression. Thus, we identified NRF1 as a positive regulator of miR‐30d in PAH and demonstrated the functional role of NRF1 in regulating PASMC proliferation and migration mediated by miR‐30d.

Sildenafil is a highly selective and potent inhibitor of PDE5A, which has been potentially used in clinical drug therapies for PAH.^[^ ^49^ ^]^ Increasing number of clinical trials have demonstrated the beneficial effect of sildenafil to improve exercise capacity and hemodynamic parameters in PAH patients.^[^ ^50^, ^51^, ^52^ ^]^ In the present study, we noticed that miR‐30d could directly target PDE5A in PAH, which prompted us to further explore whether miR‐30d could be involved in the protective effect of sildenafil against PAH. Based on miR‐30d KO rats, we observed that suppressing miR‐30d could partially attenuate the therapeutic effect of sildenafil in reducing pulmonary hypertension and pulmonary vascular remodeling in vivo, indicating an important role of miR‐30d in mediating the effect of sildenafil in PAH. Mechanistically, we further demonstrated that treatment with sildenafil induced an upregulation of both NRF1 and miR‐30d, accompanied with downregulation of miR‐30d downstream targets MTDH and PDE5A in MCT‐induced rat PH model and PDGF‐bb stimulated hPASMC. Moreover, higher content of miR‐30d mediated the protective effect of sildenafil in inhibiting hPASMC proliferation and migration. Emerging evidence has shown that sildenafil is effective to attenuate several cardiovascular diseases via regulating miRNAs. For example, sildenafil treatment was associated with downregulation of miR‐214 in chronic alcohol consumption‐induced myocardial injury.^[^ ^53^ ^]^ It has been well known that sildenafil is a selective inhibitor of PDE5A thus leading to an increased intracellular level of cyclic guanosine monophosphate (cGMP). Interestingly, cGMP was previously identified to be an activator of PGC1α which may further promote NRF1 transcriptional activity.^[^ ^54^, ^55^ ^]^ Here, we for the first time demonstrate that sildenafil can upregulate NRF1 and miR‐30d in the PASMC and provide evidence that NRF1 can directly bind to the promoter region of miR‐30d. We hypothesize that the sildenafil treatment may probably promote NRF1 transcriptional activity through the cGMP/PGC1α pathway although it needs to be further investigated. Moreover, we provide compelling evidence that miR‐30d, at least in part, contributes to the effect of sildenafil in treating PAH, and our findings of PDE5A as a direct target gene of miR‐30d may be used as a novel strategy to inhibit PDE5A.

In conclusion, miR‐30d appears to play a crucial role in regulating PAH. Our study shows a potent protective effect of miR‐30d against PAH and pulmonary vascular remodeling through regulating PASMC functions via targeting MTDH and PDE5A. We further link miR‐30d to a clinically prescribed PAH drug sildenafil and prove that miR‐30d modulates the beneficial effect of sildenafil in treating PAH. Indeed, increasing miR‐30d may be a prospective therapeutic strategy to reduce pulmonary vascular remodeling and treat PAH.

## 4 Experimental Section

### Human Plasma Samples

All human investigations conformed to the principles outlined in the Declaration of Helsinki and were approved by the institutional review committees of Tongji Hospital and Shanghai University (ECSHU 2023‐003). A total of 8 female patients with idiopathic PAH versus 10 female healthy controls were recruited with written informed consents. The diagnosis of PAH was confirmed by mean pulmonary arterial pressure (mPAP) ≥25 mmHg measured by right heart catheterization. Healthy controls are the individuals who undergo health check. Plasma samples were collected and stored at −80 °C until further examination of circulating miR‐30d levels.

### Animal Models

Male adult wild‐type (WT) rats (Charles River Labs, 200–250 g) were used to induce PH model. The miR‐30d transgenic (TG) and knockout (KO) rats were used as we previously reported.^[^ ^17^ ^]^ Briefly, rats received a single subcutaneous injection of MCT (60 mg/kg) according to previous study.^[^ ^56^ ^]^ For sildenafil treatment, WT or miR‐30d KO rats received daily gavage administration of sildenafil (1 mg/kg/d) for 28 days. At 28 days after MCT injection, right ventricular systolic pressure (RVSP) and right ventricular (RV) hypertrophy were measured. Especially, RV hypertrophy was determined by calculating the Fulton index, a ratio of the RV weight over the left ventricular (LV) plus septum (S) weight (RV/LV+S). Animal studies were approved by the Committee for the Ethics of Animal Experiments of Shanghai University (201600023) and in accordance with the Guidelines on the Care and Use of Laboratory Animals for biomedical research published by the National Institutes of Health (No. 85‐23, revised 1996).

### Hemodynamic Measurements for Right Ventricular Systolic Pressure

At 28 days after MCT injection, rats were maintained in anesthesia and ventilated after endotracheal intubation. RVSP was measured using an open‐chest technique. A 24‐gauge needle attached to pressure transducer was inserted directly into the RV and pressure curves were recorded by PowerLab (ADInstruments, USA).

### Histology Analysis of RV Hypertrophy and Pulmonary Vascular Remodeling

For histology analysis of RV hypertrophy, 5 µm‐thick cryosections of RV heart tissues were stained with wheat germ agglutinin (WGA, Sigma L4895a) to determine myocardial cross‐sectional area. For histology analysis of pulmonary vascular remodeling, 5 µm‐thick lung tissues were deparaffinized and rehydrated followed by heating with 0.01M sodium citrate (pH 6.0) and incubation with 0.3% H_2_O_2_. Lung tissue sections were then blocked and incubated with α‐SMA primary antibody (Bioworld, BS70000) and corresponding secondary antibody. The α‐SMA staining was finally visualized using diaminobenzidine (DAB). Staining images were photographed under a microscope (Leica DM3000, Germany). The number of muscularized distal pulmonary arterials per field was determined in 10–50 µm pulmonary vessels. The medial wall thickness was determined in 50–150 µm pulmonary vessels by calculating (External diameter − Internal diameter)/(External diameter) × 100%.

### Pulmonary Arterial Smooth Muscle Cell (PASMC) Culture and Treatment

The human PASMC (hPASMC) was purchased from the Cell Bank of National Collection of Authenticated Cell Cultures (Shanghai, China) and maintained in smooth muscle cell medium (SMCM, Sciencell 1101) supplemented with 2% fetal bovine serum, 1% smooth muscle cell growth factors, and 1% penicillin‐streptomycin solution. For in vitro modeling, hPASMC was starved in serum‐free SMCM and then treated with 30 ng ml^−1^ of PDGF‐bb (Sino, 10572‐H07Y) for 48h. To investigate the function of miR‐30d, hPASMC was transfected with miR‐30d mimic, inhibitor or negative control (NC) for 48h using Lipofectamine (Invitrogen). Function‐rescue experiment between miR‐30d and MTDH (or PDE5A) was performed by co‐transfection of hPASMC with miR‐30d mimic and MTDH (or PDE5A) overexpression (OE) plasmid under PDGF‐bb stress. The hPASMC was transfected with NRF1 siRNA (si‐NRF1), CREB1 siRNA (si‐CREB1), or negative control (NC‐siRNA) for 48h to examine their potential regulation of miR‐30d expression. Function‐rescue experiment between NRF1 and miR‐30d was performed by co‐transfection with si‐NRF1 and miR‐30d mimic in PDGF‐stressed hPASMC. To study the contribution of sildenafil in regulating NRF1 and miR‐30d, hPASMC was treated with sildenafil (10 µm, Selleck S4684) for 24 h and transfected with miR‐30d inhibitor for 48h under PDGF‐bb stress. At the endpoint of cell treatment, hPASMC was harvested for determinations of cell proliferation and migration and further molecular analysis. To study the function of miR‐30d under hypoxic condition, hPASMC was cultured in a 3% O_2_ chamber for 48h and transfected with miR‐30d mimic or negative control as described above.

For determination of miR‐30d expression in extracellular vesicles (EV) derived from PASMC, rat PASMC cell line (Shanghai Hexu Biotechnology Co., Ltd, HX0148) were maintained in SMCM (Sciencell 1101) supplemented with 5% fetal bovine serum, 1% smooth muscle cell growth factors, and 1% penicillin‐streptomycin solution. Rat PASMC was starved in serum‐free SMCM and then treated with 30 ng ml^−1^ of PDGF‐bb (Sino, 10572‐H07Y) in serum‐free medium for 48h before isolation of EV from the culture medium.

### Pulmonary Arterial Endothelial Cell (PAEC) Culture and Treatment

The human PAEC (hPAEC) (Shanghai Hexu Biotechnology Co., Ltd, Hum‐008) were maintained in endothelial cell medium (ECM, Sciencell 1001) supplemented with 5% fetal bovine serum, 1% endothelial cell growth supplement, and 1% penicillin‐streptomycin solution. For in vitro modeling, hPAEC was cultured in ECM under conditions of normoxia (21% O_2_) versus hypoxia (3% O_2_) for 48h. To investigate the function of miR‐30d, hPAEC was transfected with miR‐30d mimic or negative control (NC) for 48h using Lipofectamine (Invitrogen). At the endpoint of cell treatment, hPAEC was harvested for determinations of cell proliferation and apoptosis and further molecular analysis.

### Cell Proliferation and Apoptosis Analysis

To evaluate the proliferation of hPASMC, EdU (5‐ethynyl‐2’‐deoxyuridine (EdU) staining) staining was performed using kFluor488 Click‐iT EdU Kit (KeyGEN BioTECH, KGA331‐1000). Briefly, hPASMC was incubated with EdU working solution A for 24h before harvest. Cells were fixed with 4% paraformaldehyde (PFA) solution for 30 min at room temperature followed by washing with phosphate buffer solution (PBS). After incubation with 0.5% Triton X‐100 for 20 min and 5% bovine serum albumin (BSA) for 1h, cells were added with Click‐iT reagent according to manufacturer's instructions. Nuclei were counterstained with Hoechst. Images were photographed under a fluorescence microscope (Leica DMi8, Germany). The ratio of EdU‐positive hPASMC to total cell number was calculated for analysis of hPASMC proliferation. Similar procedures were used to determine EdU‐positive hPAEC as described above. TUNEL staining (Vazyme, A111‐01) was performed to determine the apoptosis of hPAEC upon hypoxic stress. The ratio of TUNEL‐positive hPAEC to total cell number was calculated for analysis of hPAEC apoptosis.

### Scratch Wound‐Healing Assay

The scratch wound‐healing assay was performed in control, PDGF‐bb, or hypoxia‐stressed hPASMC to investigate the effect of miR‐30d on cell migration. Briefly, a 200 µL pipette tip was used to scratch the cells in a straight line. Then SMCM culture medium was used to wash and remove the cell debris after scratch process. The wound at 0 h was photographed as the internal control and then cells were cultured and treated for 48 h. The wound at 48 h was photographed again at the same field. Image J software was used to analyze the area of scratch. Finally, the area at 48h divided by the area at 0h was calculated and compared between different groups.

### Luciferase Reporter Assays

To analyze the direct binding of miR‐30d to 3’UTR of MTDH and PDE5A, the wild‐type (WT) sequences and mutated (MUT) sequences of 3’UTR of MTDH (position 287–294, position 1569–1576) or PDE5A (position 26–32) were cloned into the luciferase reporter PGL3‐basic vector (Promega). HEK293T cells were transfected with luciferase reporter plasmids and miR‐30d mimic (or NC mimic), and a dual‐luciferase reporter assay was performed according to manufacturer's instructions (Promega). The luciferase reporter assays were also performed in hPASMC under PDGF‐bb stress as described above.

### ChIP‐PCR Experiment

Chromatin immunoprecipitation‐polymerase chain reaction (ChIP‐PCR) assay was performed to determine the enrichment of NRF1 in the promoter region of miR‐30d in rat PASMC cell line (Shanghai Hexu Biotechnology Co., Ltd, HX0148) using SimpleChIP Enzymatic Chromatin IP Kit (Cell Signaling Technology, 9003) according to manufacturer's instructions. The DNA fragments were prepared as previously reported,^[^ ^57^ ^]^ which were then incubated with NRF1 antibody (Abclonal, A5547) and rabbit IgG (Cell Signaling Technology, 2729). Immunoprecipitation (IP) samples were incubated with Protein G Magnetic Beads and the precipitants after centrifugation were washed and added with ChIP Elution Buffer followed by heating at 65°C overnight. The purified DNA was finally examined by quantitative real‐time‐polymerase chain reactions. The primer sequences used were as follows (5’‐3’): Forward: ATCCACACCACACAAGAATA; Reverse: GGCAAATTCTTCTTAATTGATT.

### Western Blot

Lung tissue, hPASMC, or hPAEC was lysed with RIPA lysis buffer (Beyotime, China) complemented with 1% phenylmethylsulfonyl fluoride (PMSF) and protease and phosphatase inhibitor (Beyotime, China). Equal quantity of total protein was loaded and separated by SDS‐PAGE gel and then transferred onto polyvinylidene fluoride (PVDF) membranes. The membranes were blocked with 5% non‐fat milk and incubated with primary antibody for CDK6 (Affinity, DF6448), PCNA (Abclonal, A12427), NFATC4 (Abclonal, A17511), MTDH (Abclonal, A5887), PDE5A (Abclonal, A6831), Bax (Abclonal, A12009), Bcl2 (Abclonal, A19693), NRF‐1 (Abclonal, A5547), and β‐actin (Abclonal, AC026). After incubation with corresponding secondary antibody, the immunoblots were visualized using enhanced chemiluminescence (ECL) kit and measured by ImageJ software. β‐actin was used as an internal control.

### Quantitative Real‐Time‐Polymerase Chain Reactions

Total RNA was extracted from lung tissue or hPASMC using TRIzol RNAiso Plus Kit (TaKaRa) and reverse‐transcribed to cDNA using RevertAid First Strand cDNA Synthesis Kit (Thermo K1622). Quantitative real‐time‐polymerase chain reactions (RT‐PCR) was performed using TaKaRa SYBR Premix Ex Taq (Tli RNaseH Plus, Japan) on Roche LightCycler480 PCR System. β‐actin was used as an internal control. The primers used were listed in Table S1 (Supporting Information). For miR‐30d analysis, the expression was determined using Bulge‐Loop^TM^ miRNA qRT‐PCR Primer Set for miRNA (RiboBio, Guangzhou, China) on Roche LightCycler480 PCR System. 5s was used as an internal control. For determination of circulating miR‐30d level of PAH patients and healthy controls, total RNA was extracted from plasma samples using miRVana miRNA Isolation Kit (Ambion, USA). The Caenorhabditis elegans miR‐39 (celmiR‐39) was added and served as a spike‐in control. The circulating miR‐30d expression was determined using Bulge‐Loop miRNA qRT‐PCR Primer Set as described above. Additionally, human plasma‐derived EV were isolated using size exclusion chromatography (SEC) followed by qEV original columns (35 nm, SP5102099, Izon). Rat PASMC‐derived EV were isolated from the culture medium using modified serial ultracentrifugation method.^[^ ^58^ ^]^ The EV pallets were suspended in 100 µl PBS for immediate use. Total RNA was isolated from EV for further determination of miR‐30d expression using Bulge‐Loop miRNA qRT‐PCR Primer Set for miRNA (RiboBio, Guangzhou, China). The relative expression levels of genes and miR‐30d were calculated using the 2^– ΔΔCt^ method.

### Statistical Analysis

All data were analyzed by SPSS 18.0 or GraphPad Prism 8 software. There was no pre‐processing of data in this manuscript. For human demographic and clinical characteristics, continuous variables with normal distribution are shown as means ± standard deviation (SD); continuous variables not with normal distribution are shown as median with interquartile range (IQR). Categorical variables are shown as counts (n) and percentage (%). Demographic and clinical data between 2 groups were compared using unpaired Student's *t*‐test (two sided) and Wilcoxon rank sum test as appropriate. Comparisons of the online single‐cell RNA sequencing data (accession number GSE210248) from the isolated pulmonary arteries of idiopathic PAH patient lungs versus donor lungs were performed using Wilcoxon rank sum test. For other experiments, data are shown as means ± SD. The sample size (n) was detailed in each Figure Legend. Normal distribution and equal variance assumption of all data were determined before statistical testing. For data with normal distribution, unpaired Student's *t*‐test (two‐sided) was used for comparison between 2 groups. One‐way ANOVA test followed by Bonferroni or Dunnett T3 multiple comparison test was performed for comparisons among 3 groups. Two‐way ANOVA test with Tukey correction was performed for comparisons among 4 groups. For data that did not pass normal distribution test, statistical difference was analyzed by the Mann‐Whitney U test, Kruskal‐Wallis test with the original FDR method of Benjamini and Hochberg, or robust two‐way ANOVA test followed by post‐hoc pairwiseMedianTest in the rcompanion package as appropriate. A *P* value < 0.05 was considered as statistically significant.

## Conflict of Interest

The authors declare no conflict of interest.

## Supporting information

Supporting Information

## Data Availability

The data that support the findings of this study are available from the corresponding author upon reasonable request.
