# Supplementary material for: miR‐30d Attenuates Pulmonary Arterial Hypertension via Targeting MTDH and PDE5A and Modulates the Beneficial Effect of Sildenafil
Source: Adv Sci (Weinh). 2024 Aug 29;11(40):2407712. doi: 10.1002/advs.202407712 (PMC11516105; doi:10.1002/advs.202407712)
Supplement: Supplementary file 1 — Supporting Information [file ADVS-11-2407712-s001.pdf]

ADVANCED SCIENCE
Open Access

Supporting Information

for *Adv. Sci.*, DOI 10.1002/advs.202407712

miR-30d Attenuates Pulmonary Arterial Hypertension via Targeting MTDH and PDE5A and Modulates the Beneficial Effect of Sildenafil

Xuchun Liang, Jingwen Zhou, Hongyun Wang, Ziyi Zhang, Mingming Yin, Yujiao Zhu, Lin Li, Chen Chen, Meng Wei, Meiyu Hu, Cuimei Zhao, Jianhua Yao, Guoping Li, Anh-Tuan Dinh-Xuan, Junjie Xiao* and Yihua Bei**

Supplementary Materials

Supplemental Tables

Table S1. Primer sequences for qRT-PCR.

Gene	Primer sequence
rno-CDK1-Forward	CATGGATTCTTCGCTCGT
rno-CDK1-Reverse	TGCCAGTTTGATTGTTCCT
rno-CDK6-Forward	CTGTCGTCCAGCCAGAG
rno-CDK6-Reverse	TAGAAGGGTCCTCAGCCA
rno-CCNA2-Forward	CTGAGAATGGAGCACCTTG
rno-CCNA2-Reverse	TTTCTACCTTGCAGTTGGC
rno-ANP-Forward	GAGCAAATCCCGTATACAGTGC
rno-ANP-Reverse	ATCTTCTACCGGCATCTTCTCC
rno-BNP-Forward	CTGCTTGCGGAGGCGAGAC
rno-BNP-Reverse	TGTTCTGGAGACTGGCTAGGACTTC
rno-MTDH-Forward	GTGGGATGGTAGCCGTAA
rno-MTDH-Reverse	CTCTTCTGCTGGTGCATTC
rno-PDE5A-Forward	CAACGGATAGCAGAACTCG
rno-PDE5A-Reverse	GGCGTCTATGAACCCAAC
rno-NRF1-Forward	CCAAACCCAACCCTGTC
rno-NRF1-Reverse	ACATTCTCCAAAGGTGCTG
rno-CREB1-Forward	TTCCACTTCTGCCCTCAC
rno-CREB1-Reverse	GGTCTCCTCATGGTTCCTG
rno-β-actin-Forward	AGCTGAGAGGGAAATCGTGC
rno-β-actin-Reverse	TGGACAGTGAGGCCAGGATA
hsa-CDK1-Forward	TTACAAAGATCAAGGGCTGTC
hsa-CDK1-Reverse	ACTCTGACCAAGGCATAAGAA
hsa-CDK6-Forward	CATACCCTCTCTGCTGCTTT
hsa-CDK6-Reverse	TGCTACTCATTTGCTCACCT
hsa-CCNA2-Forward	CATGTCACCGTTCCTCCT
hsa-CCNA2-Reverse	GGGCATCTTCACGCTCT
hsa-MTDH-Forward	TCTGCTGATCCCAACTCTG
hsa-MTDH-Reverse	GCTCCCTCTCCCTTTTCTT
hsa-PDE5A-Forward	AAGCATGGCTGGACGATCAC
hsa-PDE5A-Reverse	AGGCCGGTCAAATTCAGAGG
hsa-NRF1-Forward	TGCGTTGAGCTACTGACAA
hsa-NRF1-Reverse	TCTCTGACCCACCCCAT
hsa-CREB1-Forward	TTTTGTTGCCTTTGTAGCC
hsa-CREB1-Reverse	ACTTTTCTATTGGGTCCATGA

hsa-SNAI1-Forward	ACATCCGAAGCCACACG
hsa-SNAI1-Reverse	TGGGGACAGGAGAAGGG
hsa-BNIP3-Forward	AAAACAGACTTGGGTTGTGG
hsa-BNIP3-Reverse	AGGAATCACCTTGGGAAGA
hsa-LIMS1-Forward	AATTGGAAGGAGGGGTGT
hsa-LIMS1-Reverse	CAACAAAGCAGCAGCAAC
hsa-BDNF-Forward	CGTCTACCCACACGCTTC
hsa-BDNF-Reverse	CACAGCACAGCCCTTCTT
hsa-CCNE2-Forward	AACTATTTGGCTATGCTGGAG
hsa-CCNE2-Reverse	AGTGTTTTCCTGGTGGTTTT
hsa-MAPK8-Forward	TCTCCAACACCCGTACATC
hsa-MAPK8-Reverse	CCTCCAAGTCCATAACTTCCT
hsa-NOTCH1-Forward	CCGCCTTTGTGCTTCTG
hsa-NOTCH1-Reverse	GCCGCTTCTTCTTGCTG
hsa-β-actin-Forward	CACCATTGGCAATGAGCGGTTC
hsa-β-actin-Reverse	AGGTCTTTGCGGATGTCCACGT

Table S2. Demographic and clinical characteristics of patients with idiopathic pulmonary arterial hypertension (PAH) *versus* healthy controls.

	Control (n=10)	PAH (n=8)	*P* value
**Age (years)**	30±4.57	34.13±5.57	0.103
**SBP (mmHg)**	116.7±13.87	106±11.17	0.096
**DBP (mmHg)**	68.2±9.55	67.13±7.36	0.797
**BMI (kg/m^2)**	21.26±2.83	22.44±3.6	0.447
**FPG (mmol/L)**	4.87 (4.80, 5.34)	4.85 (4.56, 5.26)	0.671
**TC (mmol/L)**	5.09±1.72	4.03±0.66	0.119
**TG (mmol/L)**	1.20 (1.12, 1.78)	1.02 (0.76, 2.29)	0.500
**HDL-C (mmol/L)**	1.24±0.31	1.07±0.29	0.229
**LDL-C (mmol/L)**	2.66 (2.35, 3.33)	2.90 (2.02, 3.17)	0.812
**BUN (mmol/L)**	4.14±0.85	6.14±2.02	0.028
**Cr (µmol/L)**	52.70±7.26	60.75±12.87	0.112
**mPAP (mmHg)**		47.88±12.32	
**PVR (Wood units)**		8.74±5.86	
**PCWP (mmHg)**		7.38±2.13	
**PAH therapies**			
Tritherapies, n (%)		5 (62.5)	
Dual-therapies, n (%)		2 (25.0)	
Monotherapy, n (%)		1 (12.5)	
**Medical history**			
Hypertension, n (%)		1 (12.5)	
Diabetes, n (%)			
Hyperlipidemia, n (%)			
Atrial fibrillation, n (%)		1 (12.5)	

SBP, systolic blood pressure; DBP, diastolic blood pressure; BMI, body mass index; FPG, fasting plasma glucose; TC, total cholesterol; TG, triglyceride; HDL-C, high-density lipoprotein; LDL-C, low-density lipoprotein; BUN, blood urea nitrogen; Cr, creatinine; mPAP, mean pulmonary arterial pressure; PVR, pulmonary vascular resistance; PCWP, pulmonary capillary wedge pressure. PAH patients with tritherapies received endothelin receptor antagonists (ERA), prostacyclin analogues, and phosphodiesterase-5 (PDE-5) inhibitor (or guanylate cyclase agonist). Patients with dual-therapies received ERA and PDE-5 inhibitor (or guanylate cyclase agonist). Patients with monotherapy received ERA.

Supplemental Figures

Figure S1

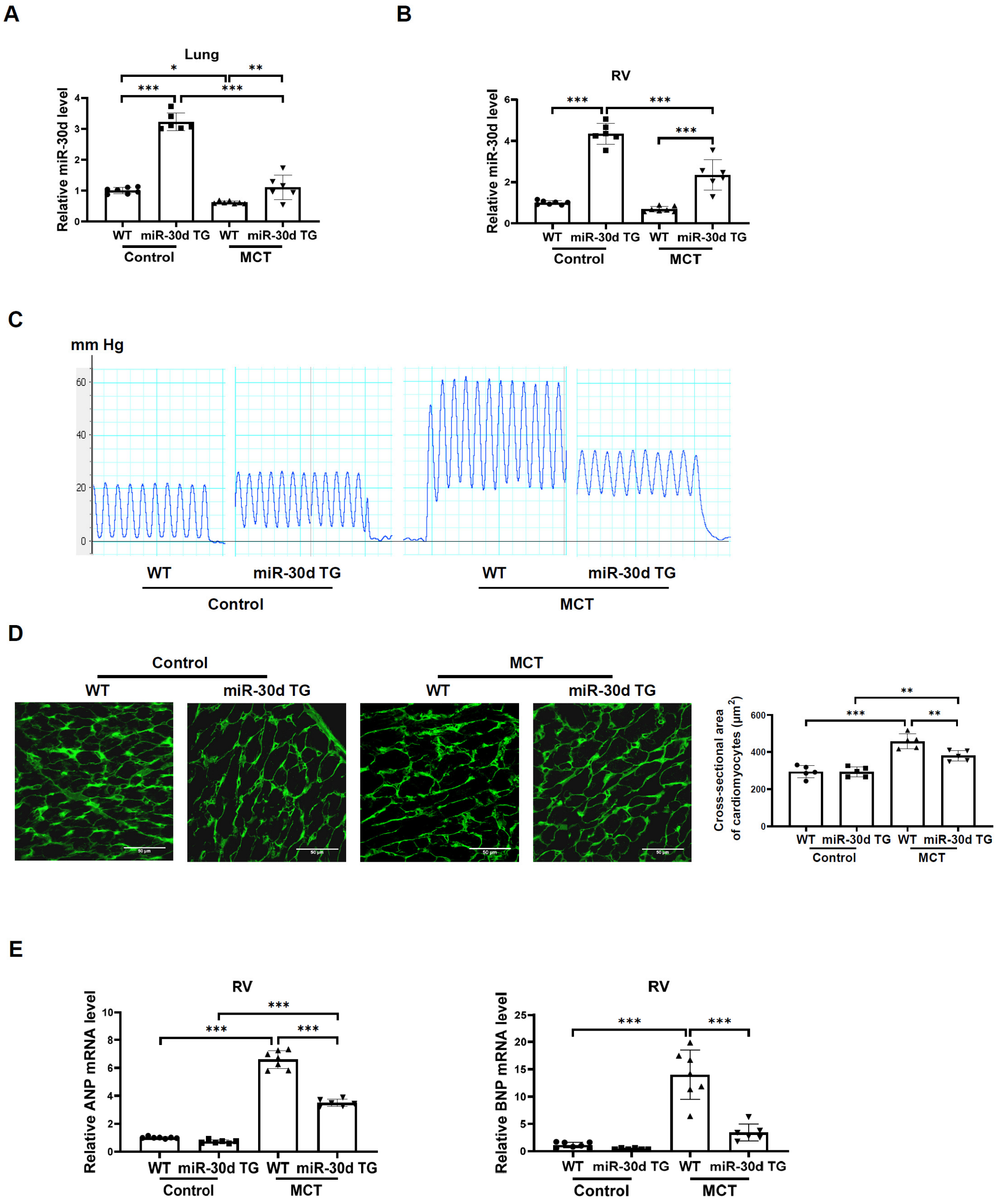

Figure S1. MiR-30d overexpression efficiency in miR-30d transgenic (TG) rats upon monocrotaline (MCT)-induced pulmonary hypertension *in vivo*. (**A and B**) qRT-PCR for miR-30d levels in lung (**A**) and right ventricle (RV) (**B**) tissues (n=6-7). (**C**) Representative images of right ventricular systolic pressure (RVSP) of rats with MCT-induced pulmonary hypertension (PH) or not as recorded by PowerLab. (**D**) Wheat germ agglutinin (WGA) staining of RV tissues (n=5). Scale bar=50 μm. (**E**) qRT-PCR for *ANP* and *BNP* mRNA levels in RV tissues (n=6-7).

Data are shown as means ± SD. Data among 4 groups were compared by robust two-way ANOVA test followed by post-hoc pairwiseMedianTest using the rcompanion package for *ANP* in (**E**), and by two-way ANOVA test followed by Tukey post hoc test for data in other figures. *, $P<0.05$; **, $P<0.01$; ***, $P<0.001$.

Figure S2

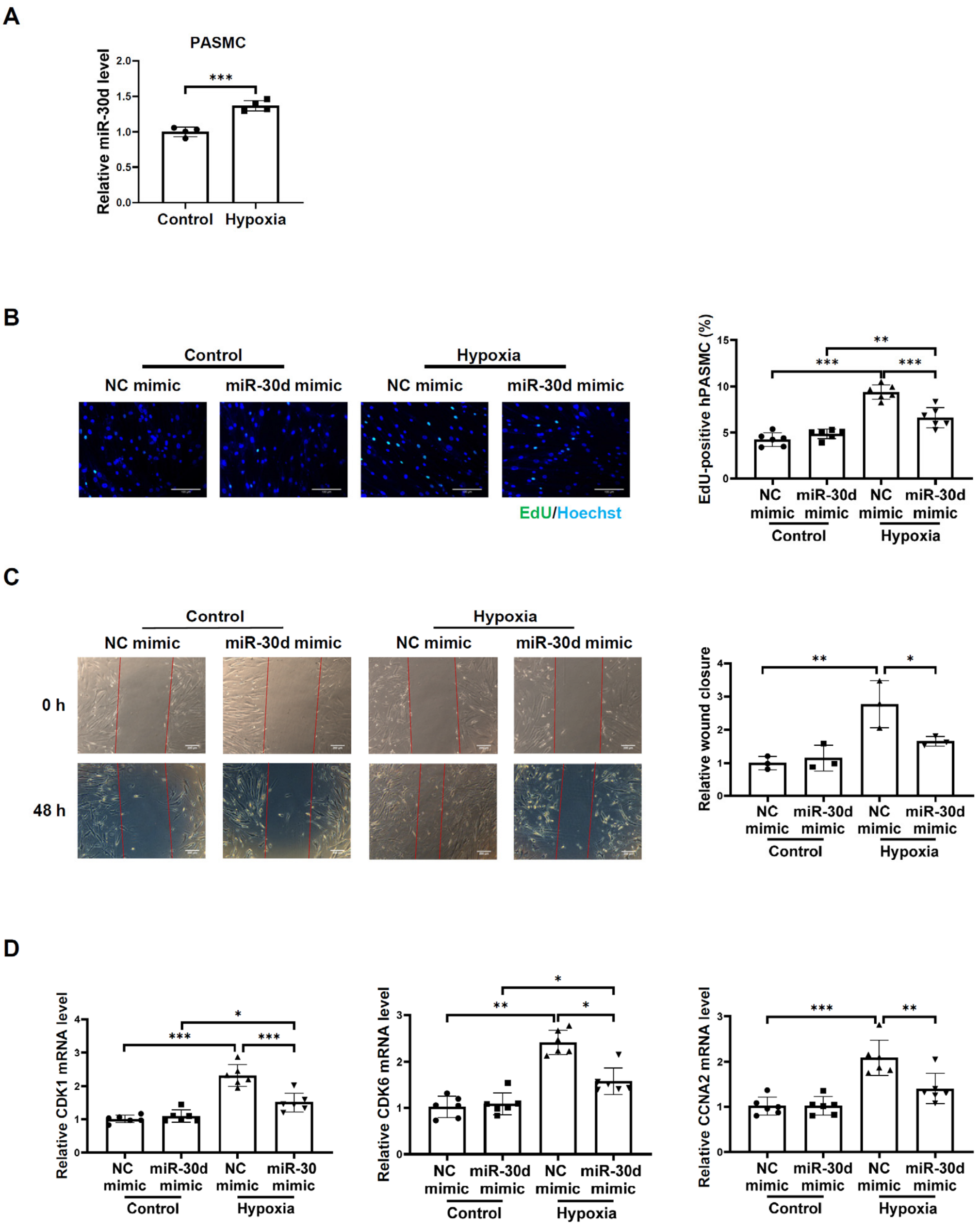

Figure S2. Overexpressing miR-30d inhibits hPASMC proliferation and migration in the hypoxic condition. (**A**) Relative miR-30d expression level in human pulmonary arterial smooth muscle cells (hPASMC) cultured in 3% hypoxic condition for 48h (n=4). (**B**) Representative images and quantification of EdU/Hoechst staining of miR-30d mimic or negative control (NC) transfected hPASMC in normoxic or 3% hypoxic condition for 48h (n=6). Scale bar=100 μm. (**C**) Scratch wound healing assay was performed to investigate the effect of miR-30d mimic on hPASMC migration. Representative images and relative wound closure of miR-30d mimic or NC transfected

hPASMC in normoxic or 3% hypoxic condition for 48h (n=3). Scale bar=200 μm. (**D**) qRT-PCR for *CDK1*, *CDK6*, and *CCNA2* mRNA levels in hPASMC (n=6).

Data are shown as means ± SD. Data between 2 groups were compared by independent-sample two-tailed Student's t-test for (**A**). Data among 4 groups were compared by robust two-way ANOVA test followed by post-hoc pairwiseMedianTest using the rcompanion package for *CDK6* in (**D**), and by two-way ANOVA test followed by Tukey post hoc test for data in other figures. *, $P<0.05$; **, $P<0.01$; ***, $P<0.001$.

Figure S3

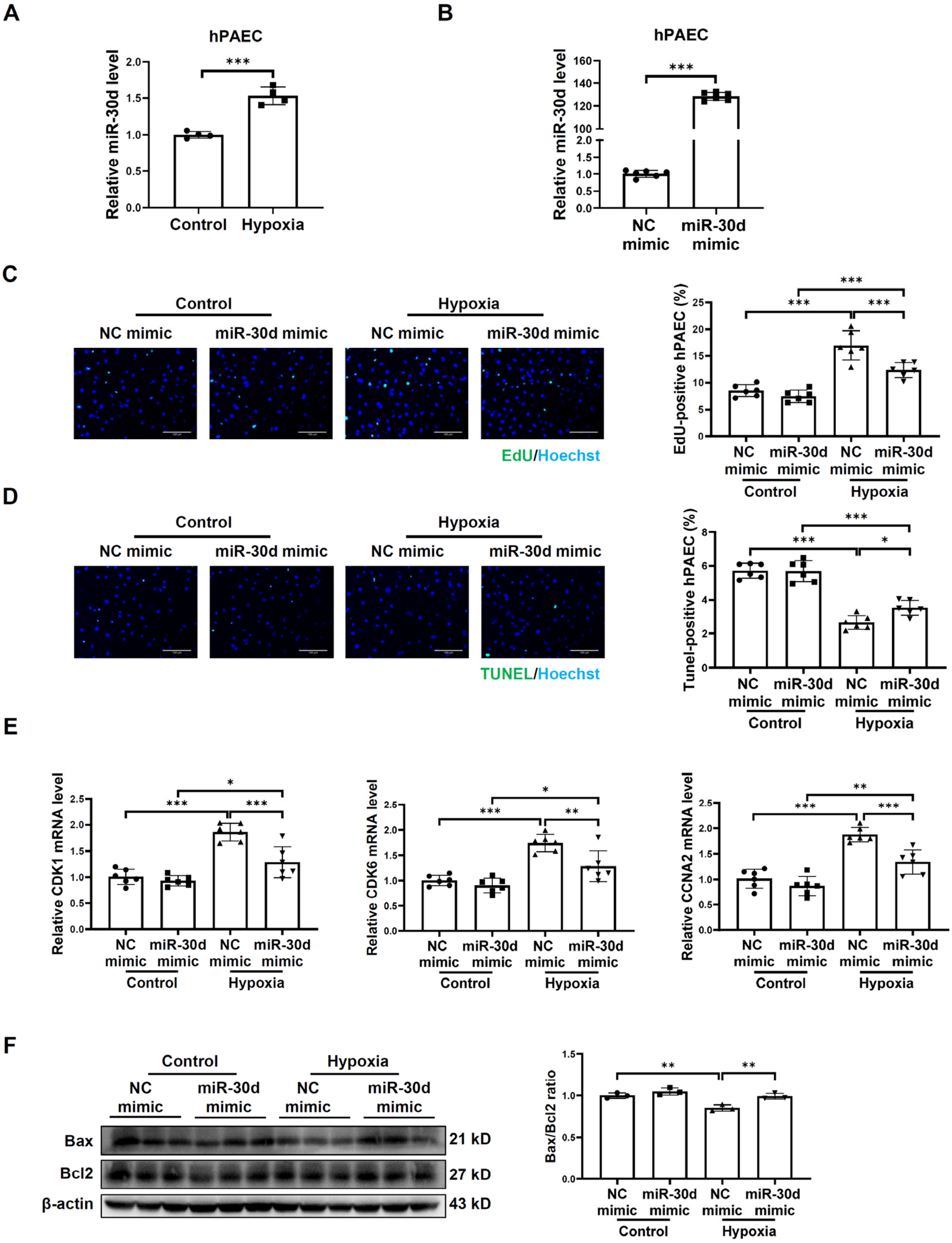

Figure S3. Overexpressing miR-30d inhibits hPAEC proliferation and its resistance to apoptosis in the hypoxic condition. (**A**) Relative miR-30d expression level in human pulmonary arterial endothelial cells (hPAEC) cultured in 3% hypoxic condition for 48h (n=4). (**B**) The overexpression efficiency of miR-30d mimic in hPAEC (n=6). (**C**) Representative images and quantification of EdU/Hoechst staining of miR-30d mimic or negative control (NC) transfected hPAEC in normoxic or 3%

hypoxic condition for 48h (n=6). Scale bar=100 μm. (**D**) Representative images and quantification of Tunel staining of miR-30d mimic or NC transfected hPAEC in normoxic or 3% hypoxic condition for 48h (n=6). Scale bar=100 μm. (**E**) qRT-PCR for *CDK1*, *CDK6*, and *CCNA2* mRNA levels in hPAEC (n=6). (**F**) Western blot for Bax and Bcl2 in hPAEC (n=3).

Data are shown as means ± SD. Data between 2 groups were compared by independent-sample two-tailed Student's t-test for (**A**) and (**B**). Data among 4 groups were compared by two-way ANOVA test followed by Tukey post hoc test for (**C**) to (**F**). *, P<0.05; **, P<0.01; ***, P<0.001.

Figure S4

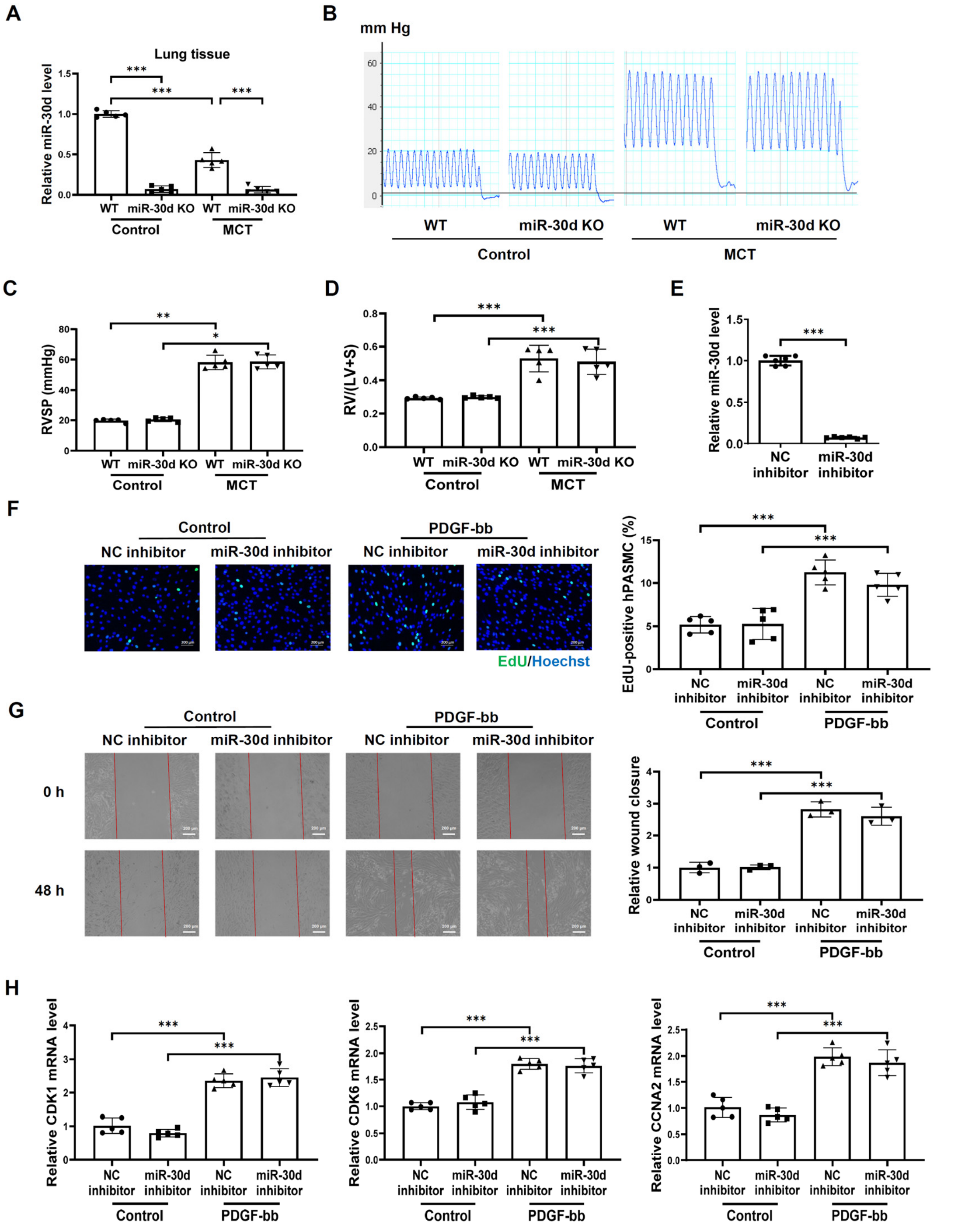

Figure S4. miR-30d knockout does not further aggravate pulmonary hypertension. (**A**) qRT-PCR for miR-30d expression level in lung tissues from wildtype (WT) and miR-30d knockout (KO) rats with monocrotaline (MCT)-induced pulmonary hypertension (PH) (n=5). (**B**) Representative images of right ventricular systolic pressure (RVSP) of rats with MCT-induced PH or not as recorded by PowerLab. (**C and D**) Right ventricular systolic pressure (RVSP) (**C**) and Fulton index (RV/(LV+S)

ratio) (**D**) were assessed in WT and miR-30d KO rats with MCT-induced PH (n=5). (**E**) The knockdown efficiency of miR-30d inhibitor in hPASMC (n=6). (**F**) Representative images and quantification of EdU/Hoechst staining of miR-30d inhibitor or negative control (NC) transfected hPASMC under PDGF-bb stress (n=5). Scale bar=200 μm. (**G**) Scratch wound healing assay was performed to investigate the effect of miR-30d inhibitor on hPASMC migration. Representative images and relative wound closure of miR-30d inhibitor or NC transfected hPASMC under PDGF-bb stress (n=3). Scale bar=200 μm. (**H**) qRT-PCR for *CDK1*, *CDK6*, and *CCNA2* mRNA levels in hPASMC (n=5).

Data are shown as means ± SD. Data between 2 groups were compared by independent-sample two-tailed Student's t-test for (**E**). Data among 4 groups were compared by robust two-way ANOVA test followed by post-hoc pairwiseMedianTest using the rcompanion package for (**C**), and by two-way ANOVA test followed by Tukey post hoc test for data in other figures. *, $P<0.05$; **, $P<0.01$; ***, $P<0.001$.

Figure S5

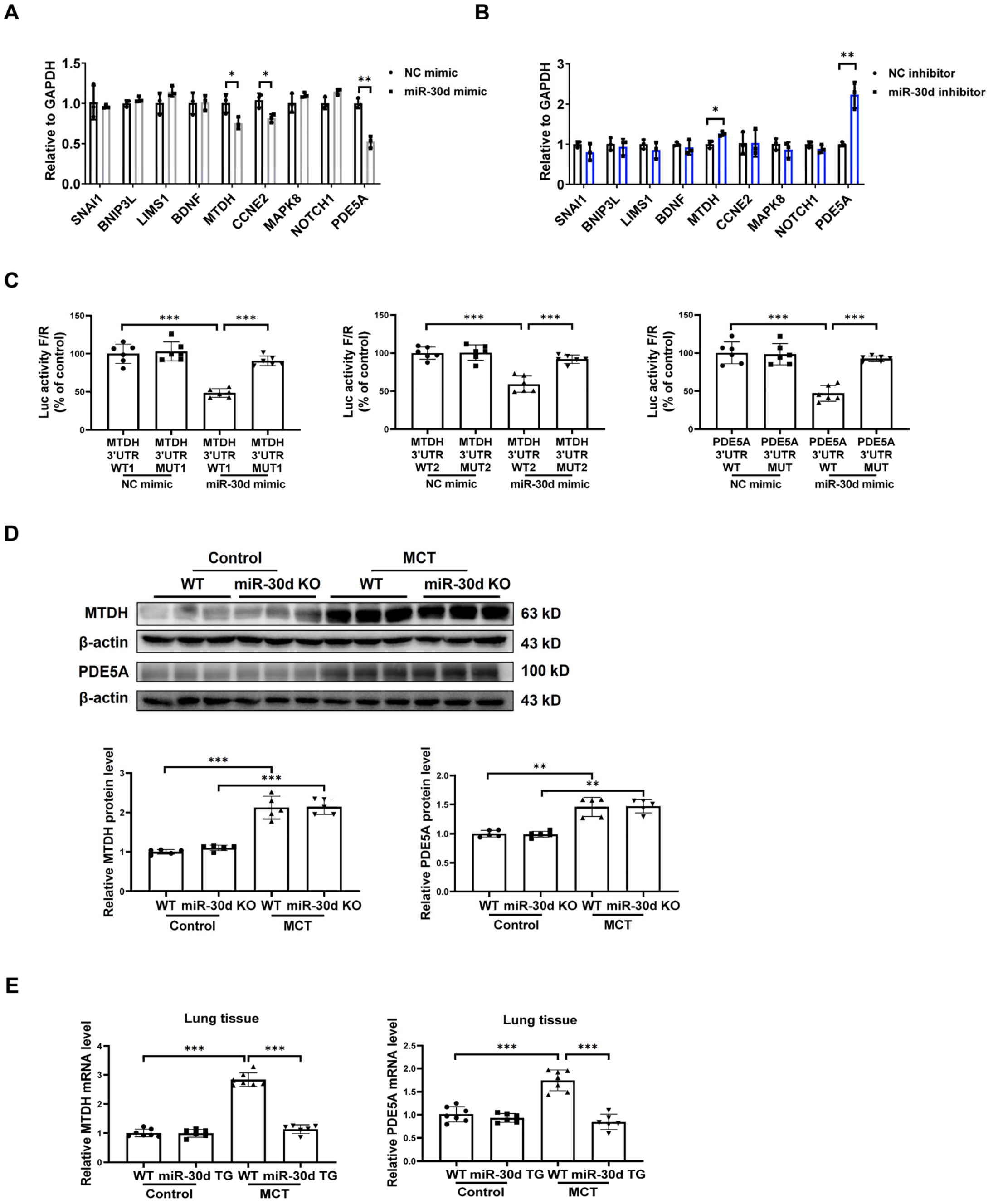

Figure S5. Identification of downstream targets of miR-30d in pulmonary arterial hypertension. (**A and B**) qRT-PCR for predicted potential target genes of miR-30d in human pulmonary arterial smooth muscle cells (hPASMC) transfected with miR-30d mimic (**A**) or inhibitor (**B**) and negative control (NC) (n=3). (**C**) Luciferase reporter assay was performed in 293T cells by transfecting the recombinant plasmids containing wild type 3'-UTR region of MTDH or PDE5A or relevant mutant sequence along with miR-30d mimic or NC mimic (n=6). (**D**) Western blot for MTDH and PDE5A in lung tissues from wild type (WT) or miR-30d knockout (KO) rats in monocrotaline (MCT)-

induced pulmonary hypertension (PH) model (n=5). (**E**) qRT-PCR for *MTDH* and *PDE5A* in lung tissues from WT or miR-30d transgenic (TG) rats in MCT-induced PH model (n=6-7).

Data are shown as means ± SD. Data between 2 groups were compared by independent-sample two-tailed Student's t-test or Mann-Whitney U test as appropriate for (**A**) and (**B**). Data among 4 groups were compared by robust two-way ANOVA test followed by post-hoc pairwiseMedianTest using the rcompanion package for PDE5A in (**D**), and by two-way ANOVA test followed by Tukey post hoc test for data in other figures. *, $P<0.05$; **, $P<0.01$; ***, $P<0.001$.

Figure S6

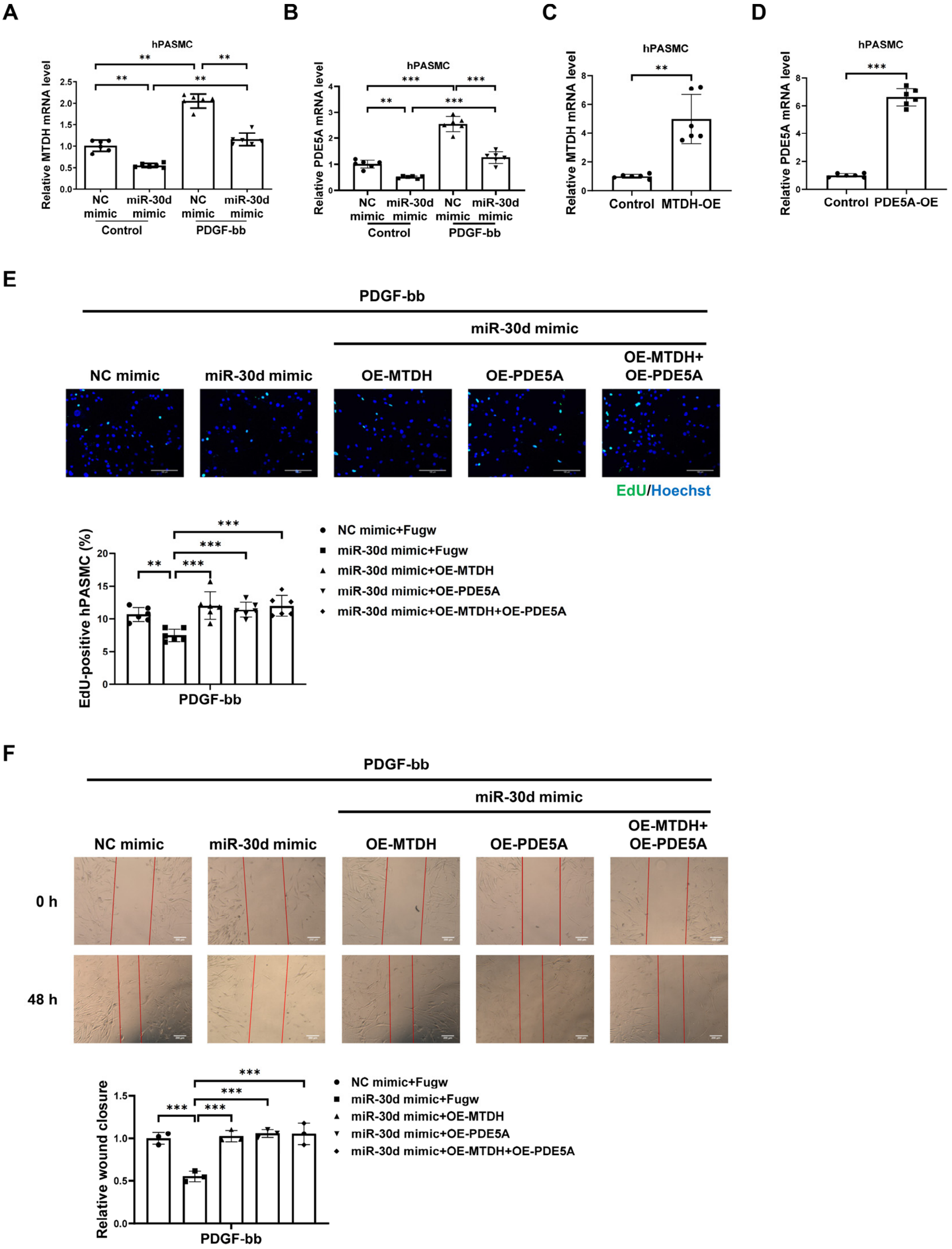

Figure S6. MiR-30d regulates human pulmonary arterial smooth muscle cells through targeting MTDH and PDE5A. (**A and B**) qRT-PCR for *MTDH* (**A**) and *PDE5A* (**B**) in human pulmonary arterial smooth muscle cells (hPASMC) transfected with miR-30d mimic or negative control (NC) after treatment of platelet-derived growth factor-bb (PDGF-bb) or not (n=6). (**C and D**) qRT-PCR for *MTDH* and *PDE5A* in hPASMC transfected with MTDH overexpression (OE) plasmid (**C**), PDE5A OE

plasmid (**D**), or control plasmid (n=6). (**E**) Representative images and quantification of EdU/Hoechst staining of hPASMC transfected with miR-30d mimic, along with MTDH- and/or PDE5A-OE plasmids after treatment with PDGF-bb (n=6). Scale bar=100 μm. (**F**) Scratch wound healing assay showing representative images and relative wound closure of hPASMC transfected with miR-30d mimic, along with MTDH- and/or PDE5A-OE plasmids after treatment with PDGF-bb (n=3). Scale bar=200 μm.

Data are shown as means ± SD. Data among 4 groups were compared by robust two-way ANOVA test followed by post-hoc pairwiseMedianTest using the rcompanion package for (**A**), and by two-way ANOVA test followed by Tukey post hoc test for (**B**). Data between 2 groups were compared by independent-sample two-tailed Student's t-test for (**C**) and (**D**). Data among 5 groups were compared by one-way ANOVA test followed by Bonferroni post-hoc test for (**E**) and (**F**). **, $P<0.01$; ***, $P<0.001$.

Figure S7

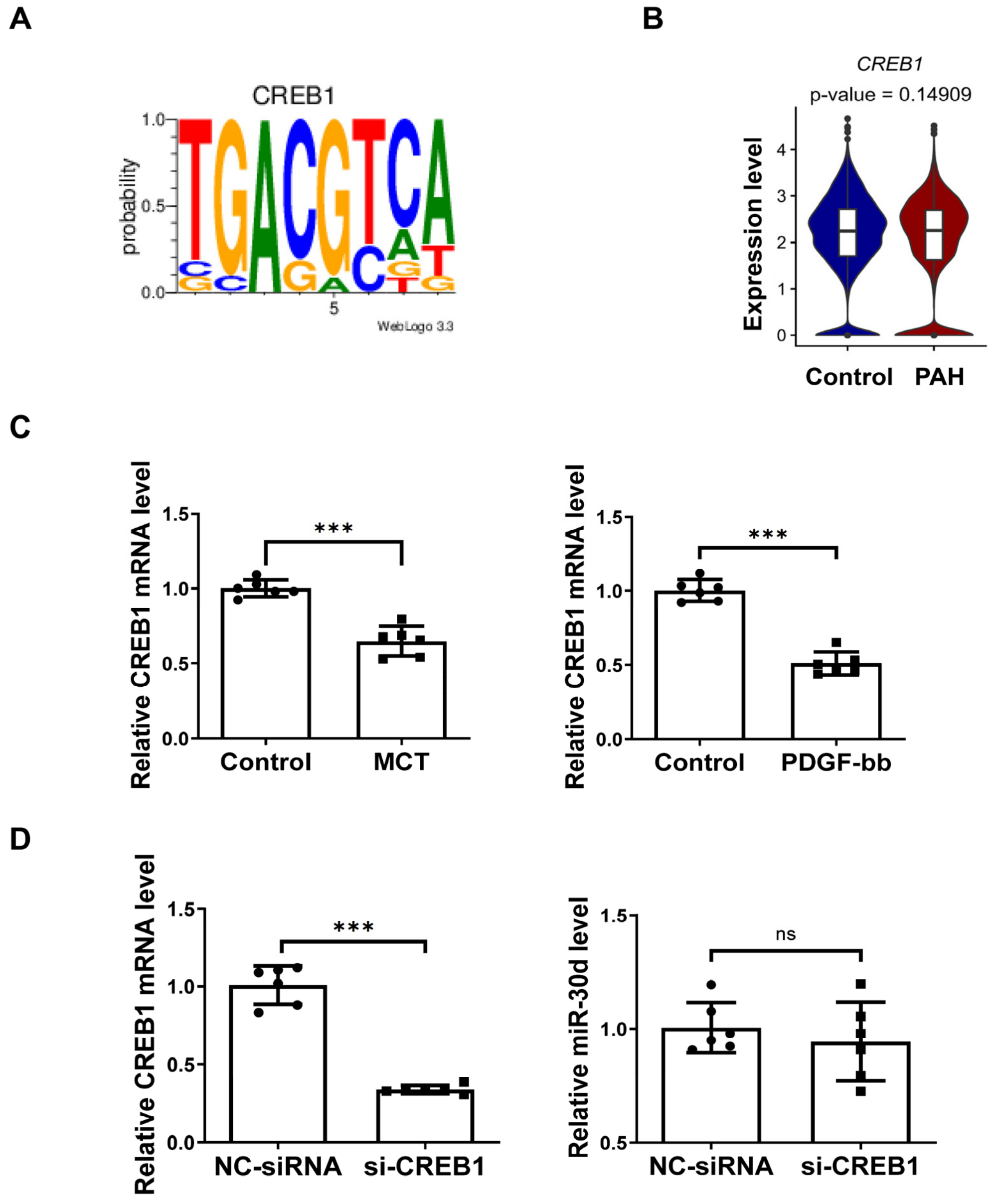

Figure S7. Expression of CREB1 in pulmonary hypertension and its regulation on miR-30d. (**A**) Predicting CREB1 as potential upstream regulators of miR-30d through DIANA TOOLS (miRGen v.3). (**B**) According to the online single cell RNA sequencing data (GEO: GSE210248) of the isolated pulmonary arteries from idiopathic pulmonary arterial hypertension (PAH) patient lungs *versus* donor lungs, comparison of CREB1 expressions in the cluster of pulmonary arterial smooth cells (PASMC) was demonstrated. (**C**) qRT-PCR analysis for *CREB1* in lung tissues from monocrotaline (MCT)-induced pulmonary hypertension (PH) model and in PDGF-treated human

PASMC (n=6). (**D**) qRT-PCR for *CREB1* and miR-30d in human PASMC transfected with CREB1 siRNA (si- CREB1) or negative control (NC-siRNA) (n=6).
Data are shown as means ± SD. Data between 2 groups were compared by independent-sample two-tailed Student's t-test for (**C**) and (**D**). ***, P<0.001; ns, not significant.

Figure S8

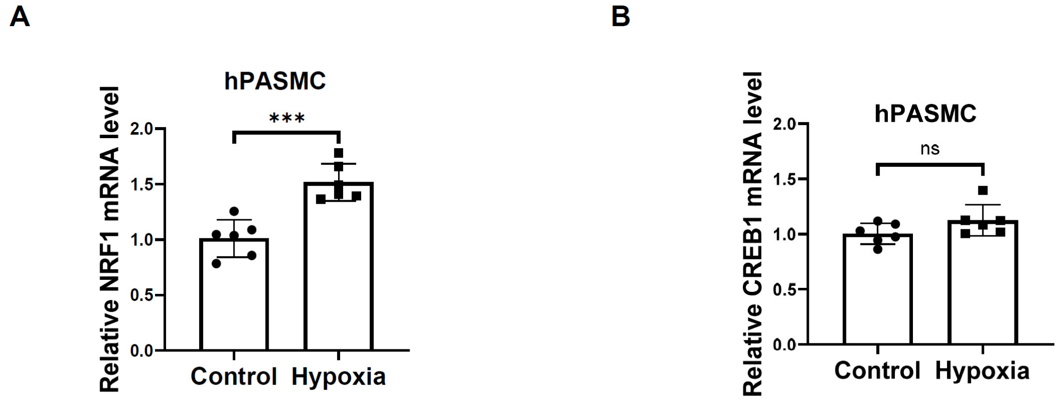

Figure S8. Regulation of NRF1 and CREB1 in human pulmonary arterial smooth muscle cells under hypoxia. (**A and B**) qRT-PCR analysis for *NRF1* (**A**) and *CREB1* (**B**) in human pulmonary arterial smooth cells (hPASMC) after normoxia (21% O_2) *versus* hypoxia (3% O_2) treatment for 48h (n=6).

Data are shown as means ± SD. Data between 2 groups were compared by independent-sample two-tailed Student's t-test for (**A**) and (**B**). ***, P<0.001; ns, not significant.

Figure S9

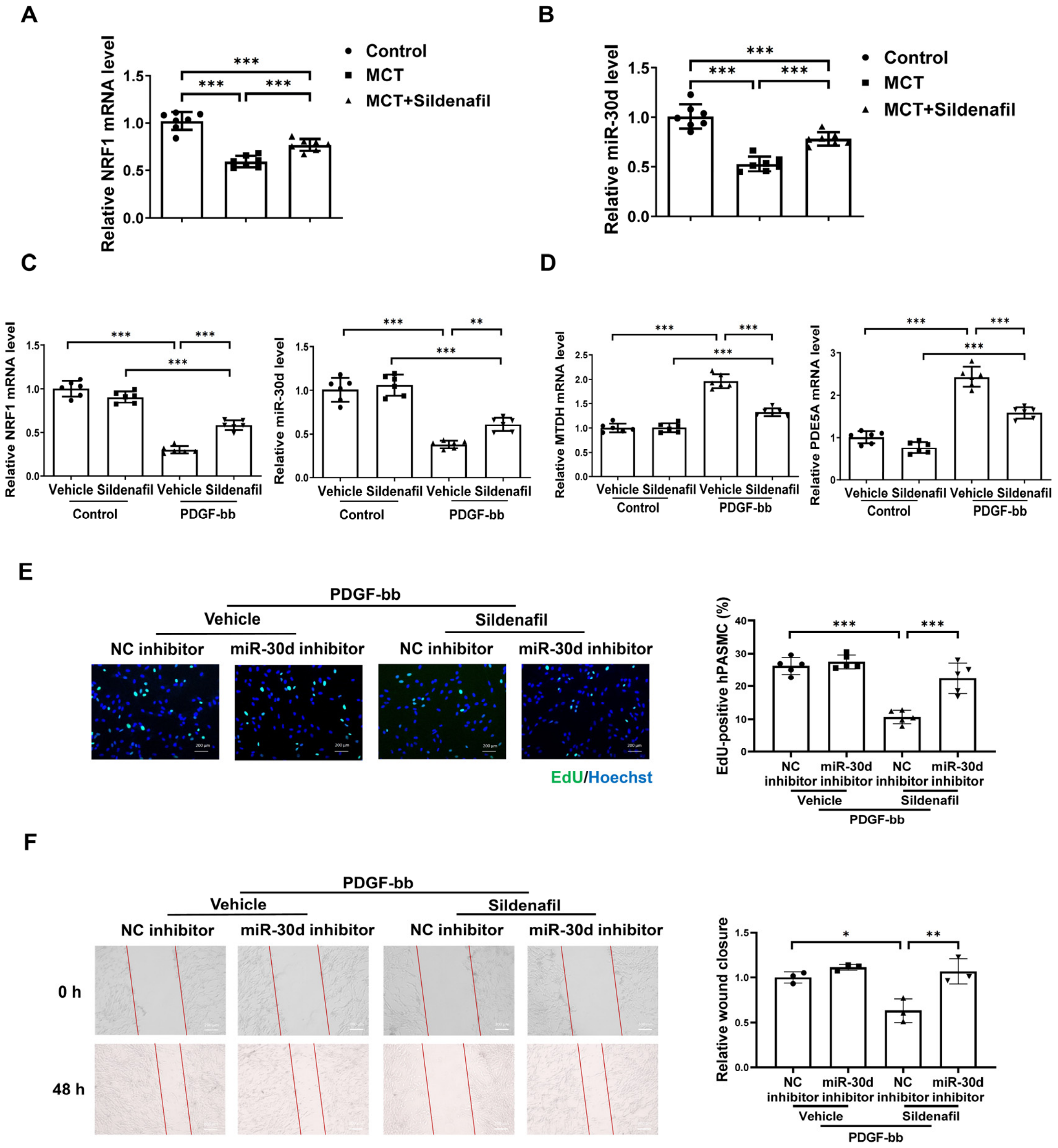

Figure S9. Sildenafil regulates NRF1 and miR-30d in pulmonary arterial hypertension. (**A and B**) qRT-PCR for *NRF1* (**A**) and miR-30d (**B**) in lung tissues from rats with sildenafil-treated monocrotaline (MCT)-induced pulmonary hypertension (PH) model (n=7). (**C and D**) qRT-PCR for *NRF1* and miR-30d (**C**) and *MTDH* and *PDE5A* (**D**) in sildenafil-treated human pulmonary arterial smooth muscle cells (hPASMC) under PDGF-bb stress or not (n=6). (**E and F**) Representative images and quantification of EdU/Hoechst staining (**E**, n=5) and relative wound closure (**F**, n=3) of miR-30d inhibitor or negative control (NC) transfected hPASMC under PDGF-bb stress treated with sildenafil or not. Scale bar=200 μm.

Data are shown as means ± SD. Data among 3 groups were compared by one-way ANOVA test followed by Bonferroni post hoc test for (**A**) and (**B**). Data among 4 groups were compared by two-way ANOVA test followed by Tukey post hoc test for (**C**) to (**F**). *, $P<0.05$; **, $P<0.01$; ***, $P<0.001$.

Figure S10

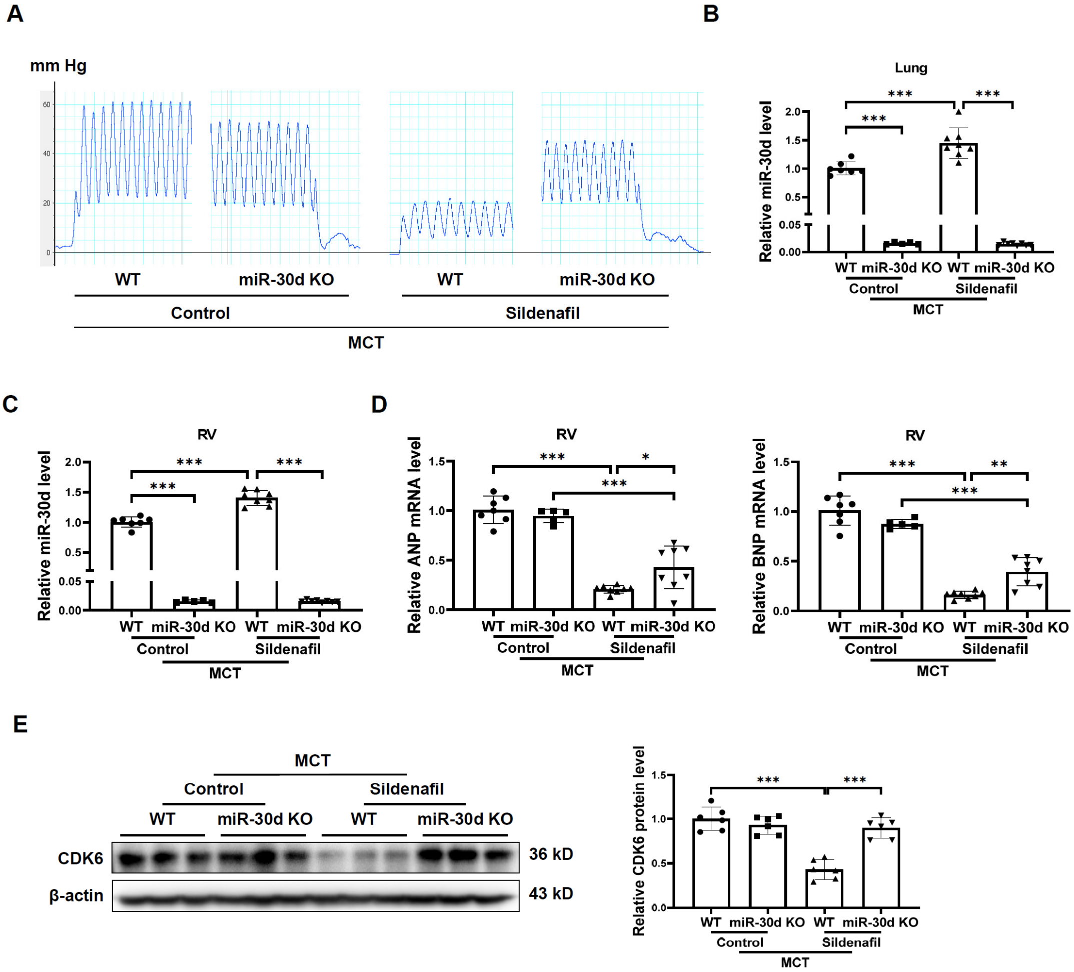

Figure S10. miR-30d and hypertrophy- and proliferation-related gene expressions in sildenafil-treated miR-30d deficiency rats with pulmonary hypertension. (**A**) Representative images of right ventricular systolic pressure (RVSP) of rats with monocrotaline (MCT)-induced pulmonary hypertension (PH) either with Sildenafil treatment or not as recorded by PowerLab. (**B and C**) qRT-PCR for miR-30d in lung (**B**) and right ventricle (RV) (**C**) tissues of wild type (WT) or miR-30d knockout (KO) rats in MCT-induced PH model treated with sildenafil or not (n=5-7 for control-treated MCT rats, n=8 for sildenafil-treated MCT rats). (**D**) qRT-PCR for *ANP* and *BNP* mRNA levels in RV tissues of WT or miR-30d KO rats in PH model treated with sildenafil or not (n=5-7 for control-treated MCT rats, n=8 for sildenafil-treated MCT rats). (**E**) Western blot for CDK6 protein level in lung tissues of WT or miR-30d KO rats in PH model treated with sildenafil or not (n=6).

Data are shown as means ± SD. Data among 4 groups were compared by two-way ANOVA test followed by Tukey post hoc test for (**B**) to (**E**). *, $P<0.05$; **, $P<0.01$; ***, $P<0.001$.
